# Supplementary material for: Use of parenteral nutrition in the first postnatal week in England and Wales: an observational study using real-world data
Source: BMJ Paediatr Open. 2022 Aug 25;6(1):e001543. doi: 10.1136/bmjpo-2022-001543 (PMC9422803; doi:10.1136/bmjpo-2022-001543)
Supplement: Supplementary data [file bmjpo-2022-001543supp001.pdf]

Online only supplemental material

eTable 1: Data fields for extraction from NNRD
eTable 2: Gestational age of neonates receiving PN in the first postnatal week as proportion of neonatal unit admissions
eTable 3: Proportion of live births receiving PN in the first postnatal week by year of birth
eTable 4: Birth weight of neonates receiving PN in the first postnatal week as proportion of live births

eTable 1: Data fields for extraction from NNRD

Outcome	
**Variable**	**Data items**
Parenteral nutrition	**PN group** defined as<br>Any of the following items entered in the 'Daily Care Fluids' and 'Feeding' during first 7 days:<br>• Y entry for PARENTERAL NUTRITION RECEIVED INDICATOR<br>Or<br>The following drug code entered in the Daily care medication during first 7 days:<br>• 1010238 Total parenteral nutrition<br><br>**No PN group** defined as<br>All other babies

Variables	
**Variable**	**Data items**
Gestational age at birth	Data extracted from GESTATION LENGTH (AT DELIVERY):
Birth weight	Data extracted from BIRTH WEIGHT
Neonatal network	Data extracted from SITE CODE (OF ACTUAL PLACE OF DELIVERY)

eTable 2: Gestational age of neonates receiving PN in the first postnatal week as proportion of neonatal unit admissions

Gestational age category† at birth	Neonates receiving PN in the first postnatal week by year of birth					
	2012	2013	2014	2015	2016	2017
Extremely preterm	2317 *(92)*	2309 *(94)*	2267 *(94)*	2348 *(94)*	2421 *(96)*	2325 *(95)*
Very preterm	3493 *(65)*	3900 *(72)*	3896 *(73)*	4059 *(74)*	4135 *(75)*	4110 *(75)*
Moderate and late preterm	2343 *(9.3)*	2640 *(10)*	2796 *(11)*	2683 *(9.7)*	2547 *(9.2)*	2375 *(8.4)*
Term	1370 *(2.9)*	1688 *(3.4)*	1618 *(3.0)*	1528 *(2.7)*	1484 *(2.5)*	1493 *(2.3)*

†Gestational age at birth categorised using WHO definitions (33)
Extremely preterm: <28^{+0} weeks, Very preterm: 28^{+0} - 31^{+6} weeks, Moderate and late preterm; 32^{+0} - 36^{+6} weeks, Term >36^{+6} weeks
Number in brackets indicates the percentage of all live births given PN in each category (Denominator data from NNRD)

eTable 3: Proportion of live births receiving PN in the first postnatal week by year of birth

Gestational age in weeks at birth	Neonates receiving PN in the first postnatal week by year of birth (%)						Trend analysis p value
	2012	2013	2014	2015	2016	2017	
<22	0.4	1.7	1.3	0.7	1.8	2.3	0.12
23	51	61	58	63	66	71.2	0.01
24	79	80	86	91	89	86	0.12
25	88	93	97	91	97	94	0.28
26	88	91	96	94	92	95	0.16
27	91	94	93	98	99	97	0.03
28	86	94	94	95	98	96	0.06
29	81	89	91	91	93	93	0.04
30	56	69	71	71	73	74	0.04
31	41	50	50	53	51	52	0.08
32	24	30	31	31	31	31	0.11
33	14	16	17	16	15	13	0.63
34	6.8	8.1	8.4	7.2	7.6	6.3	0.50
35	3.0	3.3	3.8	3.3	3.2	3.1	0.91
36	1.6	1.7	1.7	1.6	1.4	1.3	0.09
37	0.7	0.8	0.7	0.7	0.7	0.7	0.28
38	0.3	0.4	0.4	0.3	0.3	0.3	0.82
39	0.1	0.2	0.2	0.2	0.2	0.2	0.76
40	0.1	0.2	0.2	0.2	0.2	0.2	0.75
41	0.2	0.2	0.2	0.2	0.2	0.2	0.32
42	0.1	0.2	0.2	0.2	0.2	0.2	0.42

Denominator data from total number of live births from ONS Birth characteristics in England and Wales
Trend analysis for proportion of live births given PN in each category was completed: none of the trends over time were significant after Bonferroni-Holm correction for multiple comparisons
Neonates with missing data for gestational age = 3

eTable 4: Birth weight of neonates receiving PN in the first postnatal week as proportion of live births

Birth weight category* at birth	Neonates receiving PN in the first postnatal week by year of birth						Trend analysis p value
	2012	2013	2014	2015	2016	2017	
<1kg	2227 *(67)*	2578 *(72)*	2594 *(73)*	2792 *(80)*	2724 *(82)*	2533 *(100)*†	0.01
<1.5kg	5375 *(70)*	5935 *(78)*	6054 *(81)*	6341 *(84)*	6393 *(88)*	6087 *(96)*	0.00
<2.5kg	7727 *(16)*	8359 *(18)*	8444 *(18)*	8883 *(18)*	8939 *(18)*	8629 *(18)*	0.06
>2.5kg	1290 (0.2)	1550 (0.3)	1504 (0.3)	1739 (0.3)	1655 (0.3)	1674 (0.3)	0.60

**Birth weight categorised using WHO definitions (34)*
Number in brackets indicates the percentage of all live births given PN in each category (Denominator data from ONS Birth characteristics in England and Wales)
Trend analysis for proportion of neonates admitted to a neonatal unit given PN in each category: p value was significant after Bonferroni-Holm correction for multiple comparisons for <1kg and <1.5kg categories
Neonates with missing data for birth weight = 4
† The ONS changed the way they clean birthweight data in 2017 and as a result the number of neonates born <1kg dropped from 3117 births in 2016 to 2538 in 2017 This change in the denominator inflated the proportion of <1kg births given PN in 2017.
