# Supplementary material for: Use of parenteral nutrition in the first postnatal week in England and Wales: an observational study using real-world data
Source: BMJ Paediatr Open. 2022 Aug 25;6(1):e001543. doi: 10.1136/bmjpo-2022-001543 (PMC9422803; doi:10.1136/bmjpo-2022-001543)
Supplement: Author's manuscript [file bmjpo-2022-001543.draft_revisions.pdf]

BMJ Paediatrics Open

BMJ Paediatrics Open is committed to open peer review. As part of this commitment we make the peer review history of every article we publish publicly available.

When an article is published we post the peer reviewers' comments and the authors' responses online. We also post the versions of the paper that were used during peer review. These are the versions that the peer review comments apply to.

The versions of the paper that follow are the versions that were submitted during the peer review process. They are not the versions of record or the final published versions. They should not be cited or distributed as the published version of this manuscript.

BMJ Paediatrics Open is an open access journal and the full, final, typeset and author-corrected version of record of the manuscript is available on our site with no access controls, subscription charges or pay-per-view fees (http://bmjpaedsopen.bmj.com).

If you have any questions on BMJ Paediatrics Open's open peer review process please email info.bmjpo@bmj.com

BMJ Paediatrics Open

Use of parenteral nutrition in the first postnatal week in England and Wales: An observational study using real-world data

Journal:	*BMJ Paediatrics Open*
Manuscript ID	bmjpo-2022-001543
Article Type:	Original research
Date Submitted by the Author:	11-May-2022
Complete List of Authors:	Webbe, James; Imperial College London, Academic Neonatal Medicine<br>Battersby, Cheryl; Imperial College London, Medicine<br>Longford, Nicholas ; Imperial College London, Department of Medicine<br>Oughham , Kayleigh; Imperial College London Faculty of Medicine, Neonatal Data Analysis Unit<br>uthaya, s; Imperial College London, Neonatal Unit<br>Modi, Neena; Imperial College London, Neonatal Medicine<br>Gale, Chris; Imperial College London, Academic Neonatal Medicine
Keywords:	Neonatology, Epidemiology

SCHOLARONE™
Manuscripts

I, the Submitting Author has the right to grant and does grant on behalf of all authors of the Work (as defined in the below author licence), an exclusive licence and/or a non-exclusive licence for contributions from authors who are: i) UK Crown employees; ii) where BMJ has agreed a CC-BY licence shall apply, and/or iii) in accordance with the terms applicable for US Federal Government officers or employees acting as part of their official duties; on a worldwide, perpetual, irrevocable, royalty-free basis to BMJ Publishing Group Ltd ("BMJ") its licensees and where the relevant Journal is co-owned by BMJ to the co-owners of the Journal, to publish the Work in this journal and any other BMJ products and to exploit all rights, as set out in our licence.

The Submitting Author accepts and understands that any supply made under these terms is made by BMJ to the Submitting Author unless you are acting as an employee on behalf of your employer or a postgraduate student of an affiliated institution which is paying any applicable article publishing charge ("APC") for Open Access articles. Where the Submitting Author wishes to make the Work available on an Open Access basis (and intends to pay the relevant APC), the terms of reuse of such Open Access shall be governed by a Creative Commons licence – details of these licences and which Creative Commons licence will apply to this Work are set out in our licence referred to above.

Other than as permitted in any relevant BMJ Author's Self Archiving Policies, I confirm this Work has not been accepted for publication elsewhere, is not being considered for publication elsewhere and does not duplicate material already published. I confirm all authors consent to publication of this Work and authorise the granting of this licence.

Confidential: For Review Only

Use of parenteral nutrition in the first postnatal week in England and Wales: An observational study using real-world data

J Webbe[1], C Battersby[2], N Longford[3], K Ougham[4], S Uthaya[5], N Modi[6], C Gale[7]

[1] James Webbe, PhD

Neonatal Medicine, School of Public Health, Faculty of Medicine, Imperial College London

j.webbe@imperial.ac.uk

[2] Cheryl Battersby, PhD

Neonatal Medicine, School of Public Health, Faculty of Medicine, Imperial College London

c.battersby@imperial.ac.uk

[3] Nicholas Longford, PhD

Neonatal Medicine, School of Public Health, Faculty of Medicine, Imperial College London

n.longford@imperial.ac.uk

[4] Kayleigh Ougham, MSc

Neonatal Medicine, School of Public Health, Faculty of Medicine, Imperial College London

k.ougham@imperial.ac.uk

[5] Sabita Uthaya, MD

Neonatal Medicine, School of Public Health, Faculty of Medicine, Imperial College London

s.uthaya@imperial.ac.uk

[6] Neena Modi, FMedSci

Confidential: For Review Only

Neonatal Medicine, School of Public Health, Faculty of Medicine, Imperial College London

n.modi@imperial.ac.uk

[7] Chris Gale, PhD

Neonatal Medicine, School of Public Health, Faculty of Medicine, Imperial College London

christopher.gale@imperial.ac.uk

Corresponding author

Dr Christopher Gale, Reader in Neonatal Medicine

Neonatal Medicine, Chelsea and Westminster campus, Imperial College London

369 Fulham Road, London, SW10 9NX, United Kingdom.

+ 44 (0)203 3155418

christopher.gale@imperial.ac.uk

Manuscript word count: 2252

Confidential: For Review Only

ABSTRACT

Background

Parenteral nutrition (PN) is used to provide supplemental support to neonates while enteral feeding is being established. PN is a high-cost intervention with beneficial and harmful effects. Internationally there is substantial variation in how PN is used, and there are limited contemporary data describing use across the UK.

Objective

To describe PN use in the first postnatal week in infants born and admitted to neonatal care in England, Scotland and Wales.

Method

Data describing neonates admitted to National Health Service (NHS) neonatal units between 1st January 2012 and 31st December 2017, extracted from routinely recorded data held the National Neonatal Research Database (NNRD); the denominator was live births, from Office for National Statistics.

Results

Over the study period 62,145 neonates were given PN in the first postnatal week (1.4% of all live births); use was higher in more preterm neonates (76% of livebirths at <28 weeks, 0.2% of term livebirths) and in neonates with lower birth weight. 15% (9181/62145) of neonates given PN in the first postnatal week were born at term. There was geographic variation in PN administration: the proportion of live births given PN within neonatal regional networks ranged from 1.0% (95% confidence intervals: 1.0, 1.0) to 2.8% (95% confidence interval: 2.7, 2.9).

Conclusions and relevance

Significant variation exists in neonatal PN use; it is unlikely this reflects optimal use of an expensive intervention. Research is needed to identify which babies will benefit most and which are at risk of harm from early PN.

Registration

ClinicalTrials.gov: NCT03767634

Confidential: For Review Only

Registration date: 06/12/2018

Abstract word count: 250/250

Confidential: For Review Only

INTRODUCTION

In 1968 parenteral nutrition (PN) was used to support the metabolic needs of a term neonate with small bowel atresia (1). Following this, PN has been increasingly used to supplement the nutrition of sick or preterm neonates. The widespread use of PN has been encouraged on the basis that optimising nutrition will improve short and long-term outcomes (2). It is considered most beneficial for neonates born preterm or with lower birth weight who have fewer reserves and may accrue large nutritional deficits before enteral feeds are established (2). Despite widespread use, the impact of PN on key neonatal outcomes has not been evaluated in randomised controlled neonatal trials powered for clinically meaningful and functional end-points.

Therefore, while effects on short-term biochemical markers such as nitrogen balance are well described (3), evidence to support beneficial effects on survival and neurodevelopment are lacking (3, 4). Conversely, PN carries well described risks, of which the most serious and common is bloodstream infection (5). Recent evidence from large randomised controlled trials in critically unwell adults (6) and children (7) showed that use of PN during the first seven days of admission to an intensive care unit led to worse outcomes, when compared to delayed PN administration, indicating that the harms of early PN outweigh benefits in these populations. Although there has not been a similar trial in neonatal care, subgroup analysis in the PePaNIC trial of term neonates looked after on paediatric intensive care units also showed increased rates of nosocomial infection with early PN use (8), suggesting that early PN use should be targeted at neonates with most potential for benefit.

Given the uncertain balance of risk and benefit for neonatal PN use, it is unsurprising that international practice is variable: some neonatal units in high-income countries provide PN to up to 70% of neonatal admissions (9) while others report not using PN (10). In the United Kingdom, a 2011 report from the National Confidential Enquiry into Patient Outcome and Death found considerable variation in neonatal management in 2008 with only 24% of patients receiving PN that was considered best practice (11). Following this, a national

Confidential: For Review Only

framework (12), and NICE guidance (13) have been developed. These make recommendations about prescription, administration, monitoring and recipients of PN in neonatal units; recommending all neonates born before 30^{+0} $weeks^{+days}$ gestation or weighing under 1250g at birth, and any who are unable (or not expected) to establish milk feeds of ≥100mL/kg/day by postnatal day five, receive early PN.

We aimed to describe how PN is used in the first postnatal week, to explore how use is influenced by gestational age, birth weight, geographical region, and to compare how use has changed over time in the period prior to the publication of 2020 NICE guidance.

OBJECTIVE

To describe the pattern of PN use in neonatal units in England, Scotland and Wales in the first seven postnatal days.

METHODS

Study design

This study was an epidemiological description of practice: we pre-registered it (Clinicaltrials.gov: NCT03767634) and published the study protocol (14). We report it in line with RECORD guidelines (15).

Data source

We used de-identified data held in the National Neonatal Research Database (NNRD) (16). The NNRD holds data extracted from electronic health records completed by health professionals during routine clinical care (17). The Neonatal Data Set, a defined national data standard (18) comprising approximately 450 items, is extracted and transmitted to the Neonatal Data Analysis Unit at Imperial College London. The NNRD holds data from all neonates admitted to NHS neonatal units in England, Scotland and Wales; in total the NNRD contains data from about one million neonates from 2008 to the present. Accuracy and

Confidential: For Review Only

completeness of NNRD data have been confirmed by comparison with Case Record Forms from a prospective clinical trial, which showed high data completeness and accuracy (>95%) (19). The Chief Investigator of the NNRD (NM) holds UK Research Ethics Committee approval (REC Reference: 16/LO/1093), and Confidentiality Advisory Group approval (ECC 8-05(f/2010)) for the NNRD as a research database. Study specific Research Ethics Committee (18/NI/0214), Health Research Authority and Health and Care Research Wales approval and approval for inclusion of their data in this study from all English neonatal units was obtained. Data for this study was extracted by author KO, operating within the guidelines established by these approvals; no other investigators had accessed the wider NNRD for this study. No data cleaning methods were required for this study, and no data linkage was required. We obtained population-level data for total live births by gestational age and birth weight from the Office for National Statistics (ONS) (20-25), and for live births by neonatal network from Mothers and Babies: Reducing Risk through Audits and Confidential Enquiries across the UK (MBRRACE-UK) reports (26-29), for denominator data. Population-level data for total neonatal unit admissions by gestational age and birth weight were obtained from the NNRD for denominator data.

Confidential: For Review Only

Participants

The study population was all neonates born between 1st January 2012 and 31st December 2017 and admitted to a neonatal unit in England and Wales. There were no exclusion criteria: this was intended to maximise the sample size and ensure complete, population-level data.

Variables

The primary outcome was any use of PN in the first seven postnatal days. To describe the background characteristics of neonates given PN we extracted data relating to gestational age at birth, birth weight, year of birth, and neonatal network (30) of birth. The variables extracted from the NNRD, and how they were defined, are listed in eTable 1.

Statistical methods

We described the characteristics of neonates that received PN in the first seven postnatal days and compared use between different groups. For gestational age, we grouped neonates by completed weeks of gestation according to WHO categories (31). For birth weight, we grouped neonates according to WHO categories (32). To explore differences in PN use across geographical regions, we grouped neonates by neonatal network of birth (30). We compared changes in PN prescribing over time by grouping neonates according to the year of birth.

We used the Student's t-test to test differences in rates of PN among different groups, and a linear regression t-test to test for a change in PN use within different categories over time. We corrected for multiple comparisons using the Bonferroni-Holm method (33, 34).

RESULTS

The only deviation from the protocol related to the use of data from neonatal units in Scotland. We were unable to use data on babies from Scottish neonatal units due to difficulties obtaining the institutional approvals required for Public Benefit and Privacy Panel for Health and Social Care (35) approval. The project was completed using data from neonatal units in England and Wales only.

Over the six-year study period 4,196,314 neonates were born in England and Wales. Of these, 347,959 neonates were admitted to NHS neonatal units and had data held within the NNRD; 62,145 were recorded as having received PN during the first postnatal week. We did not identify a significant difference over time in the proportion of neonates that received PN: just over 1% of all live births (Table 1) and 17% of all neonatal unit admissions (eTable 2). PN was given to neonates of all gestational ages, with neonates born preterm more likely to be recipients. However, a large proportion of the neonates who receive PN were more mature: 15% of neonates who received PN in the first postnatal week over the study period were born at term. We did not find a significant change in PN use over this period in any gestational age category after correction for multiple comparisons (Table 1).

https://mc.manuscriptcentral.com/bmjpo

Confidential: For Review Only

Table 1 Gestational age of neonates receiving PN in the first postnatal week as a proportion of total live births

Gestational age category* at birth	Neonates receiving PN in the first postnatal week by year of birth n (% of live births)						Trend analysis p value
	2012	2013	2014	2015	2016	2017	
Extremely preterm	2317 *(72)*	2309 *(75)*	2267 *(77)*	2348 *(78)*	2421 *(76)*	2325 *(75)*	0.14
Very preterm	3493 *(61)*	3900 *(71)*	3896 *(71)*	4059 *(73)*	4135 *(73)*	4110 *(74)*	0.03
Moderate and late preterm	2343 *(5.3)*	2640 *(6.1)*	2796 *(6.4)*	2683 *(6.0)*	2547 *(5.6)*	2375 *(5.2)*	0.52
Term	1370 *(0.2)*	1688 *(0.3)*	1618 *(0.3)*	1528 *(0.2)*	1484 *(0.2)*	1493 *(0.2)*	0.04
Total	9523 *(1.3)*	10537 *(1.5)*	10577 *(1.5)*	10618 *(1.5)*	10587 *(1.5)*	10303 *(1.5)*	0.02

**Gestational age at birth categorised using WHO definitions (31)*
Extremely preterm: <28^{+0} weeks, Very preterm: 28^{+0} - 31^{+6} weeks, Moderate and late preterm; 32^{+0} - 36^{+6} weeks, Term >36^{+6} weeks
Number in brackets indicates the percentage of all live births given PN in each category (Denominator data from ONS Birth characteristics in England and Wales)
Trend analysis for proportion of live births given PN in each category: none of the trends over time were significant after Bonferroni-Holm correction for multiple comparisons
Neonates with missing data for gestational age = 3

The proportion of live births who receive PN in the first postnatal week is lower in neonates born at 22 or 23 weeks, rises to over 90% for neonates born from 25 to 28 weeks gestation before falling again (Figure 1). When analysed by gestational age no change over the six years was found in PN use in the first postnatal week; no significant trend was observed after correction for multiple comparisons (eTable 3).

Neonates born with lower birth weights were more likely to receive PN in the first postnatal week (Table 2, eTable 4). A significant increase in PN administration was seen among neonates with birthweight <1.5kg over the study period.

Table 2 Birth weight of neonates receiving PN in the first postnatal week as proportion of neonatal unit admissions

Confidential: For Review Only

Birth weight at birth*	Neonates receiving PN in the first postnatal week by year of birth n (% of neonatal units admissions)						Trend analysis p value
	2012	2013	2014	2015	2016	2017	
<1kg	2401 *(92)*	2484 *(94)*	2442 *(95)*	2542 *(95)*	2547 *(96)*	2541 *(96)*	0.001
<1.5kg	5591 *(77)*	5878 *(82)*	5936 *(83)*	6090 *(84)*	6213 *(85)*	6095 *(85)*	0.01
<2.5kg	7999 *(27)*	8679 *(29)*	8756 *(29)*	8880 *(28)*	8935 *(28)*	8628 *(28)*	0.16
>2.5kg	1524 *(3.1)*	1857 *(3.5)*	1821 *(3.2)*	1738 *(2.8)*	1652 *(2.6)*	1675 *(2.4)*	0.3

**Birth weight categorised using WHO definitions (32)*
Number in brackets indicates the percentage of all neonates admitted to a neonatal unit given PN in each category (Denominator data from NNRD)
Trend analysis for proportion of neonates admitted to a neonatal unit given PN in each category: p value was significant after Bonferroni-Holm correction for multiple comparisons for <1kg and <1.5kg categories
Neonates with missing data for birth weight = 4

The use of PN differed across networks, with a range from 1.0% to 2.8% of all live births given PN in the first postnatal week (Figure 2); these were significantly different from the national average, 1.5% all live births, for nine of the 14 neonatal networks (Table 4). Rates of PN administration within neonatal networks varied less over time than the differences seen between networks.

Table 3 Neonates receiving PN in the first postnatal week by Neonatal Network as proportion of live births

Neonatal Network at birth	Babies receiving PN in the first postnatal week by year of birth n (% of live births)					
	2012	2013	2014	2015	2016	2017
East Midlands[1]	608 *(†)*	601 *(1.1%)*	607 *(1.1%)*	553 *(1.0%)*	570 *(1.0%)*	566 *(1.0%)*
East of England	953 *(†)*	973 *(1.4%)*	1052 *(1.5%)*	942 *(1.4%)*	961 *(1.4%)*	926 *(1.4%)*
London - North Central & East	771 *(†)*	733 *(1.4%)*	806 *(1.5%)*	842 *(1.6%)*	845 *(1.6%)*	845 *(1.6%)*
London - North West	417 *(†)*	429 *(1.4%)*	421 *(1.3%)*	403 *(1.3%)*	443 *(1.4%)*	409 *(1.3%)*
London - South	725 *(†)*	723 *(1.6%)*	739 *(1.7%)*	804 *(1.8%)*	828 *(1.8%)*	764 *(1.7%)*
North West	1233 *(†)*	1206 *(1.5%)*	1222 *(1.5%)*	1231 *(1.5%)*	1130 *(1.3%)*	1031 *(1.2%)*
Northern	326 *(†)*	398 *(1.2%)*	397 *(1.2%)*	408 *(1.3%)*	413 *(1.3%)*	363 *(1.1%)*

Confidential: For Review Only

South East Coast	681 (†)	723 (1.5%)	707 (1.5%)	720 (1.5%)	717 (1.5%)	710 (1.5%)
South West	849 (†)	805 (1.7%)	785 (1.6%)	839 (1.8%)	813 (1.7%)	786 (1.6%)
Southern West Midlands	425 (†)	426 (1.3%)	443 (1.4%)	431 (1.4%)	484 (1.5%)	454 (1.5%)
Staffordshire, Shropshire, & Black Country	495 (†)	534 (2.1%)	527 (2.1%)	553 (2.3%)	479 (1.9%)	499 (2.0%)
Thames Valley & Wessex	1062 (†)	1068 (1.9%)	1021 (1.7%)	1052 (1.8%)	1045 (1.7%)	1034 (1.8%)
Wales	166 (†)	933 (2.9%)	910 (2.9%)	881 (2.8%)	846 (2.7%)	888 (2.8%)
Yorkshire & Humber	692 (†)	820 (1.2%)	791 (1.2%)	795 (1.2%)	847 (1.3%)	878 (1.3%)

[1]East Midlands Operational Delivery Network previously the separate Central and Trent Networks
Number in brackets indicates the percentage of all live births given PN in each Neonatal Network (Data from MBRRACE-UK reports)
[†]MBRRACE-UK report not produced for 2012
Neonates with missing data for neonatal network = 202

DISCUSSION

In this work, we described the characteristics of neonates born in England and Wales who receive PN in the first postnatal week. PN is a common intervention and is given to 17% of all neonatal unit admissions. While PN use is higher in neonates born prematurely and with lower birth weight, a considerable proportion are born at term. We also show that use varies between different neonatal networks.

That higher rates of PN use is seen in neonates born more preterm is unsurprising, as these populations are considered most likely to benefit. The lower rates seen in the most preterm neonates (those born at <26 gestational weeks) are likely because many of these babies die before admission to a neonatal unit and before PN is commenced. However, it is noteworthy that around 15% of all babies given PN are born at term. The energy requirements (36), indications for use and metabolic stability of these groups differs, thus the risks and benefits of PN may also differ substantially across gestational ages.

Confidential: For Review Only

We found PN use in the first postnatal week varied significantly between neonatal networks. This is in keeping with variations between neonatal units in PN use in other settings (9, 10). However, we were unable to find another comprehensive population based study of national PN use. From our descriptive analysis, it is not possible to determine whether the variation we identified is explained by other case-mix factors (such as congenital anomalies or neonatal surgical conditions), nor, given the paucity of evidence, is it possible to comment on what rate of PN use should be expected. However, by identifying wide variation in practice within the United Kingdom, we highlight early PN use as an area where optimal practice is uncertain (37). This variation also has financial implications. Treating a neonate with PN for one day in 2012 cost £37.43 (38), and if all hospitals in England and Wales treated the same proportion of live births as the highest use network this would cost the NHS an additional £2.5 million each year. This expense may be justified if PN leads to lower mortality or morbidity, but such evidence is lacking.

The strengths of our study include the population-level coverage involving a cohort of over 4 million neonates. The population-level data meant that recruitment bias was reduced, but not fully eliminated. The NNRD covers all neonatal units in England and Wales but does not include Paediatric Intensive Care Units and surgical units that admit neonates. Some term cardiac and surgical babies will be cared for on these units and thus we may have underestimated the amount of PN used in these groups. We followed a pre-specified protocol and data analysis plan and limited the risk of false discovery associated with multiple comparisons (39) by using the Holm-Bonferroni method. In keeping with previous studies that have used the NNRD, we had very little missing data (19, 40). Limitations of this study include that we were not able to obtain permission to use data for neonates born in Scotland, reducing the study population. As the NNRD only holds data for neonates admitted to an NHS neonatal unit for denominator data, we used data from the ONS and MBRRACE-UK to provide total numbers of live births. As MBRRACE-UK did not produce a report in 2012 this limited the number of years for which we could undertake network level comparisons. As has been found

https://mc.manuscriptcentral.com/bmjpo

Confidential: For Review Only

in previous studies, due to the lack of additional information about PN in the data extracted we are unable to describe changes in how PN was used (e.g. when PN was commenced or types of PN used) (41-43).

Our findings show that PN use in the first postnatal week is common in England and Wales, with regional variation. In light of the potential harm found with early PN in critically unwell children (7), and the lack of evidence of benefit for clinically important endpoints in infants (3, 44) research is needed to ensure that PN use in neonates is underpinned by a robust evidence base. Our data will help in the planning of future trials to identify which neonates will benefit from early PN and ensure that clinical practice is based on strong evidence. Approximately twenty-eight neonates are started on PN each day in England and Wales: if all of these diverse patients are to receive optimal treatment urgent research is needed to ensure they are the neonates who will benefit most from this intervention, and to avoid harm in others.

CONCLUSION

Parenteral nutrition is commonly used in the first postnatal week across the United Kingdom, with higher use in neonates born more preterm. Across all gestational age categories no change in PN use in the first postnatal week over time was found, but there is persisting variation in use between regional neonatal networks. Research is needed to ensure PN use in this group is well targeted.

Confidential: For Review Only

ABBREVIATIONS

ELBW	Extremely Low Birth Weight
LBW	Low Birth Weight
MBRRACE-UK	Mothers and Babies: Reducing Risk through Audits and Confidential Enquiries across the UK
NNRD	National Neonatal Research Database
ONS	Office for National Statistics
PICU	Paediatric Intensive Care Unit
PN	Parenteral Nutrition
VLBW	Very Low Birth Weight

Confidential: For Review Only

DECLARATIONS

Consent for publication

Not applicable

Availability of data and material

Applications to use the data used within this project should be made to the Neonatal Data Analysis Unit, Imperial College London

Competing interests

JW has received support from Chiesi Pharmaceuticals to attend an educational conference and has received a research grant from Mason Medical Research Foundation.

SU has received funding from the National Institute of Health Research, the Department of Health and Prolacta Life Sciences. SU has been on the Advisory Board of Fresenius Kabi and received honoraria and travel expenses for speaking at study days organised by Fresenius Kabi. SU is a member of the National Institute for Health and Care Excellence Parenteral Nutrition Guideline Development Committee. NM is the Chief Investgator for the National Neonatal Research Database. In the last five years NM has served on the Board of Trustees of the Royal College of Paediatrics and Child Health, David Harvey Trust, Medical Women's Federation, Medact, Action Cerebral Palsy, TheirWorld and Academy of Medical Sciences. NM is a member of the Nestle Scientific Advisory Board but accepts no personal remuneration for this role. NM has received research grants from the British Heart Foundation, Medical Research Council, National Institute of Health Research, Westminster Research Fund, Prolacta Life Sciences, Chiesi, Takeda, March of Dimes, Health Data Research UK, HCA International, and European Health Data Evidence Network; travel and accommodation expenses from Prolacta, Nestle and Chiesi.

Confidential: For Review Only

CG has received support from Chiesi Pharmaceuticals to attend an educational conference; in the past 5 years he been investigator on received research grants from Medical Research Council, National Institute of Health Research, Canadian Institute of Health Research, Department of Health in England, Mason Medical Research Foundation, Westminster Medical School Research Trust and Chiesi Pharmaceuticals.

CB has received support from Chiesi Pharmaceuticals to attend an educational conference; she is supported by a personal award by the National Institute of Health Research.

Funding

The NNRD is created and maintained through grants and unrestricted funding held by NM. This includes costs involved in data transfer, storage, cleaning, merging, administration and regulatory approvals. The extraction of study data from the NNRD and analysis for this study was funded through a Mason Medical Research Fellowship awarded to JW. CG is funded by the United Kingdom Medical Research Council (MRC) through a Clinician Scientist Fellowship award. The MRC and Mason Medical Research Foundation were not involved in the design of the study, collection, analysis, and interpretation of data or in writing the manuscript

Authors' contributions

JW, CG and NM conceived this project. JW, CG and NL planned the statistical analyses. The first draft of the manuscript was written by JW and revised by NM; CG, SU, CB, NL and NM edited and reviewed the manuscript. It was approved by JW, CG, CB, NL, SU and NM.

Acknowledgements

We wish to thank Angela Richard-Löndt and Laura Noakes, parents of preterm infants, for their support in developing this research project and BLISS for their input and support developing the dissemination plan.

Confidential: For Review Only

What is already known on this topic?

- Parenteral nutrition is commonly given to neonates unable to receive adequate milk feeds or while milk feeds are introduced
- There is limited evidence to inform parenteral nutrition use in neonates

What this study adds?

- The early use of parenteral nutrition is common; 17% of all babies admitted to neonatal units receive PN in the first postnatal week
- Fifteen percent of neonates given PN in the first postnatal week are born full-term
- There is variation in the use of parenteral nutrition in the first week of postnatal life between regional neonatal networks

Confidential: For Review Only

REFERENCES

1. Wilmore DW, Dudrick SJ. Growth and Development of an Infant Receiving All Nutrients Exclusively by Vein. Jama. 1968;203(10):860-4.
2. Ziegler EE, Thureen PJ, Carlson SJ. Aggressive nutrition of the very low birthweight infant. Clinics in perinatology. 2002;29(2):225-44.
3. Trivedi A, Sinn JK. Early versus late administration of amino acids in preterm infants receiving parenteral nutrition. Cochrane Database Syst Rev. 2013(7):CD008771.
4. Moyses HE, Johnson MJ, Leaf AA, Cornelius VR. Early parenteral nutrition and growth outcomes in preterm infants: a systematic review and meta-analysis. The American journal of clinical nutrition. 2013;97(4):816-26.
5. Zingg W, Tomaske M, Martin M. Risk of parenteral nutrition in neonates--an overview. Nutrients. 2012;4(10):1490-503.
6. Casaer MP, Mesotten D, Hermans G, Wouters PJ, Schetz M, Meyfroidt G, et al. Early versus late parenteral nutrition in critically ill adults. N Engl J Med. 2011;365(6):506-17.
7. Fivez T, Kerklaan D, Mesotten D, Verbruggen S, Wouters PJ, Vanhorebeek I, et al. Early versus Late Parenteral Nutrition in Critically Ill Children. N Engl J Med. 2016;374(12):1111-22.
8. van Puffelen E, Vanhorebeek I, Joosten KFM, Wouters PJ, Van den Berghe G, Verbruggen S. Early versus late parenteral nutrition in critically ill, term neonates: a preplanned secondary subgroup analysis of the PEPaNIC multicentre, randomised controlled trial. The Lancet Child & adolescent health. 2018;2(7):505-15.
9. Christensen RD, Henry E, Wiedmeier SE, Burnett J, Lambert DK. Identifying patients, on the first day of life, at high-risk of developing parenteral nutrition-associated liver disease. Journal of perinatology : official journal of the California Perinatal Association. 2007;27(5):284-90.
10. Ksiazyk J, Swietlinski J, Brozek G, Musialik-Swietlinska E, Migdal M, Zejda JE. [Parenteral nutrition in neonatal units: a survey of current practices in Poland]. Medycyna wieku rozwojowego. 2008;12(4 Pt 1):899-904.
11. Mason DG, Puntis JWL, McCormick K, Smith N. Parenteral nutrition for neonates and children: a mixed bag. Archives of Disease in Childhood. 2011;96(3):209.
12. British Association of Perinatal Medicine. The Provision of Parenteral Nutrition within Neonatal Services - A Framework for Practice [Guideline]. 2016 [updated April 2016. Available from: http://www.bapm.org/publications/documents/guidelines/Parenteral%20Nutrition%20April%202016.pdf.
13. Excellence; NIfHaC. Neonatal parenteral nutrition: NICE guideline [NG154]. 2020.
14. Webbe J, Longford N, Uthaya S, Modi N, Gale C. Outcomes following early parenteral nutrition use in preterm neonates: protocol for an observational study. BMJ open. 2019;9(7):e029065.
15. Benchimol EI, Smeeth L, Guttmann A, Harron K, Moher D, Petersen I, et al. The REporting of studies Conducted using Observational Routinely-collected health Data (RECORD) Statement. PLOS Medicine. 2015;12(10):e1001885.
16. Gale C, Morris I. The UK National Neonatal Research Database: using neonatal data for research, quality improvement and more. Archives of disease in childhood Education and practice edition. 2016;101(4):216-8.
17. Spencer A, Modi N. National neonatal data to support specialist care and improve infant outcomes. Arch Dis Child Fetal Neonatal Ed. 2013;98(2):F175-80.
18. NHS Digital. National Neonatal Data Set 2017 [Available from: http://www.datadictionary.nhs.uk/data_dictionary/messages/clinical_data_sets/data_sets/national_neonatal_data_set/national_neonatal_data_set_-_episodic_and_daily_care_fr.asp?shownav=1.

Confidential: For Review Only

19. Battersby C, Statnikov Y, Santhakumaran S, Gray D, Modi N, Costeloe K, et al. The United Kingdom National Neonatal Research Database: A validation study. PloS one. 2018;13(8).
20. Office for National Statistics. Characteristics of Birth 1, England and Wales: 2012. Fareham: Vital Statistics Outputs Branch, Office for National Statistics; 2013.
21. Office for National Statistics. Characteristics of Birth 1, England and Wales: 2013. Fareham: Vital Statistics Outputs Branch, Office for National Statistics; 2014.
22. Office for National Statistics. Birth Characteristics in England and Wales: 2014. Fareham: Vital Statistics Outputs Branch, Office for National Statistics; 2015.
23. Office for National Statistics. Birth Characteristics in England and Wales: 2015. Fareham: Vital Statistics Outputs Branch, Office for National Statistics; 2016.
24. Office for National Statistics. Birth Characteristics in England and Wales: 2016. Fareham: Vital Statistics Outputs Branch, Office for National Statistics; 2017.
25. Office for National Statistics. Birth Characteristics, England and Wales: 2017. Fareham: Vital Statistics Outputs Branch, Office for National Statistics; 2018.
26. Draper ES, Gallimore ID, Kurinczuk JJ, Smith PW, Boby T, Smith LK, et al. MBRRACE-UK Perinatal Mortality Surveillance Report, UK Perinatal Deaths for Births from January to December 2016. Leicester: The Infant Mortality and Morbidity Studies, Department of Health Sciences, University of Leicester; 2018.
27. Manktelow BN, Smith LK, Prunet C, Smith PW, Boby T, Hyman-Taylor P, et al. MBRRACE-UK Perinatal Surveillance Report: UK Perinatal Deaths for Births from January to December 2015. Leicester: The Infant Mortality and Morbidity Studies, Department of Health Sciences, University of Leicester; 2017.
28. Manktelow BN, Smith LK, Seaton SE, Hyman-Taylor P, Kurinczuk JJ, Field DJ, et al. MBRRACE-UK Perinatal Mortality Surveillance Report, UK Perinatal Deaths for Births from January to December 2014. Leicester: The Infant Mortality and Morbidity Studies, Department of Health Sciences, University of Leicester; 2016.
29. Manktelow BN, Smith LK, Evans TA, Hyman-Taylor P, Kurinczuk JJ, Field DJ, et al. Perinatal Mortality Surveillance Report UK Perinatal Deaths for births from January to December 2013. Leicester: The Infant Mortality and Morbidity Group, Department of Health Sciences, University of Leicester; 2015.
30. Gale C, Santhakumaran S, Nagarajan S, Statnikov Y, Modi N, Unit NDA, et al. Impact of managed clinical networks on NHS specialist neonatal services in England: population based study. Brit Med J. 2012;344.
31. Blencowe H, Cousens S, Chou D, Oestergaard M, Say L, Moller AB, et al. Born Too Soon: The global epidemiology of 15 million preterm births. Reprod Health. 2013;10.
32. World Health Organization. International Statistical Classification of Diseases and Related Health Problems 10th Revision. 2016.
33. Proschan MA, Waclawiw MA. Practical Guidelines for Multiplicity Adjustment in Clinical Trials. Control Clin Trials. 2000;21(6):527-39.
34. Holm S. A Simple Sequentially Rejective Multiple Test Procedure. Scand J Stat. 1979;6(2):65-70.
35. Morris C, Gray A, Scott J, editors. NHS Scotland Public Benefit and Privacy Panel (PBPP) – Much Ado about Governance 3 years on. International Population Data Linkage Conference; 2018; Banff, Canada.
36. Schanler RJ, Garza C, Nichols BL. Fortified mothers' milk for very low birth weight infants: results of growth and nutrient balance studies. J Pediatr. 1985;107(3):437-45.
37. Soll RF, McGuire W. Evidence-Based Practice: Improving the Quality of Perinatal Care. Neonatology. 2019:1-6.
38. Walter E, Liu FX, Maton P, Storme T, Perrinet M, von Delft O, et al. Cost analysis of neonatal and pediatric parenteral nutrition in Europe: a multi-country study. European Journal Of Clinical Nutrition. 2012;66:639.
39. Young SS, Karr A. Deming, data and observational studies. Significance. 2011;8(3):116-20.

Confidential: For Review Only

40. Battersby C, Longford N, Mandalia S, Costeloe K, Modi N, group UKNCNEs. Incidence and enteral feed antecedents of severe neonatal necrotising enterocolitis across neonatal networks in England, 2012-13: a whole-population surveillance study. Lancet Gastroenterol Hepatol. 2017;2(1):43-51.
41. Gale C, Jeyakumaran D, Longford N, Battersby C, Ojha S, Oughham K, et al. Administration of parenteral nutrition during therapeutic hypothermia: a population level observational study using routinely collected data held in the National Neonatal Research Database. Archives of Disease in Childhood - Fetal and Neonatal Edition. 2021;106(6):608.
42. Uthaya S, Longford N, Battersby C, Oughham K, Lanoue J, Modi N. Early versus later initiation of parenteral nutrition for very preterm infants: a propensity score-matched observational study. Archives of Disease in Childhood - Fetal and Neonatal Edition. 2021:fetalneonatal-2021-322383.
43. Webbe JWH, Longford N, Battersby C, Oughham K, Uthaya SN, Modi N, et al. Outcomes in relation to early parenteral nutrition use in preterm neonates born between 30 and 33 weeks' gestation: a propensity score matched observational study. Arch Dis Child Fetal Neonatal Ed. 2021.
44. Simmer K, Rao SC. Early introduction of lipids to parenterally-fed preterm infants. Cochrane Database Syst Rev. 2005(2):CD005256.

Confidential: For Review Only

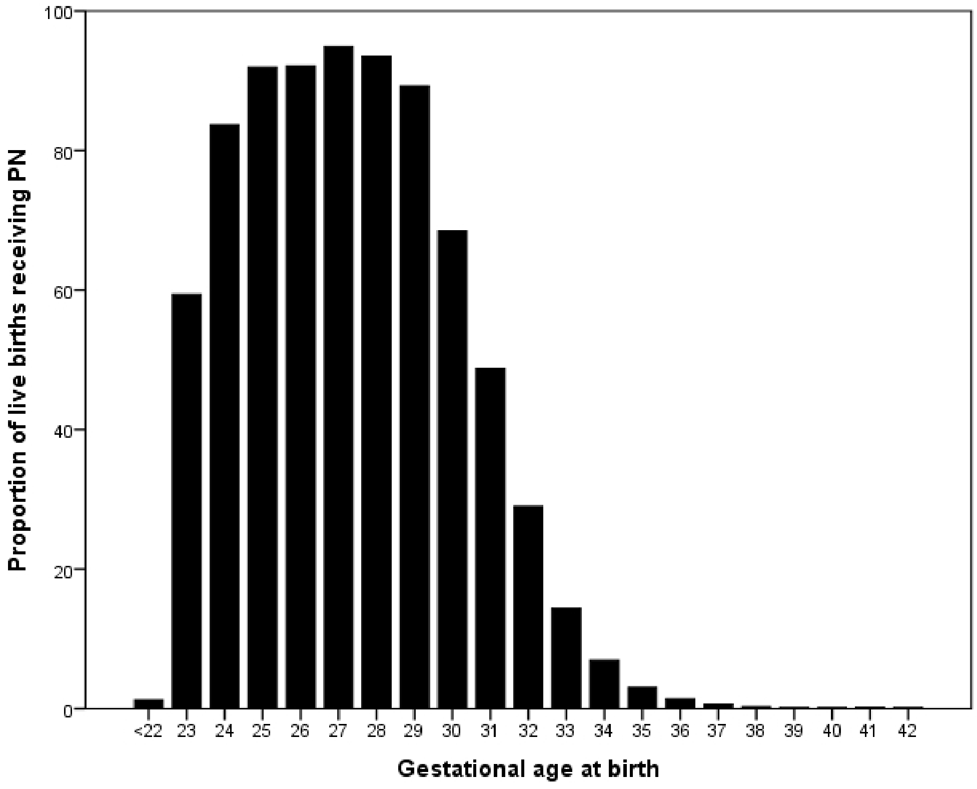

220x176mm (72 x 72 DPI)

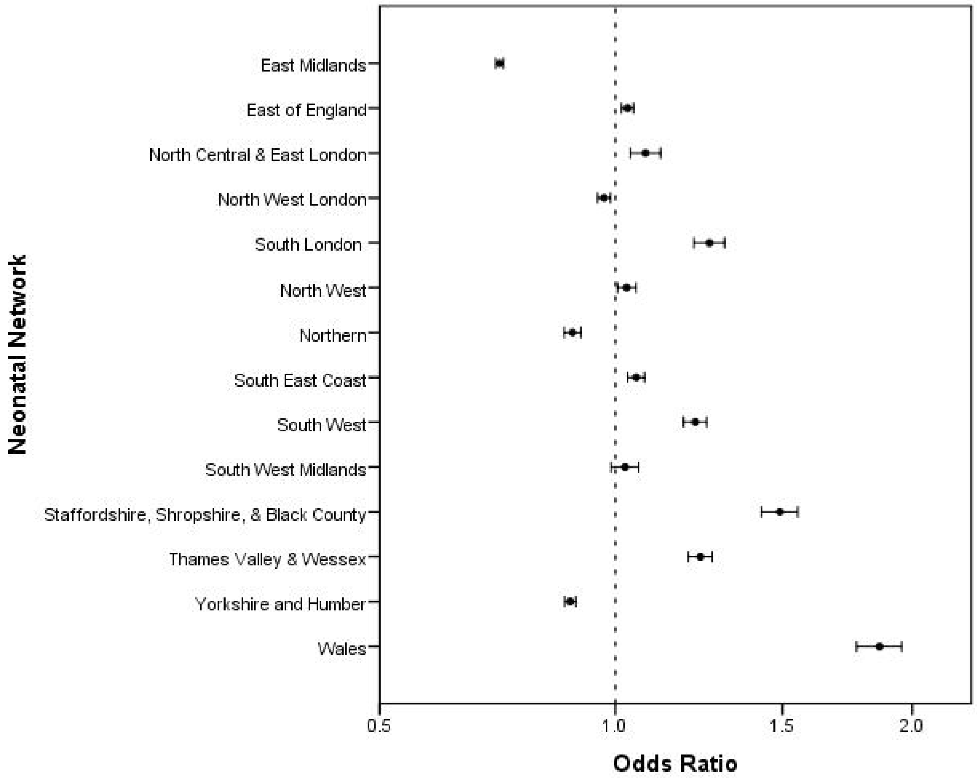

418x334mm (38 x 38 DPI)

Online only supplemental material

eTable 1: Data fields for extraction from NNRD
eTable 2: Gestational age of neonates receiving PN in the first postnatal week as proportion of neonatal unit admissions
eTable 3: Proportion of live births receiving PN in the first postnatal week by year of birth
eTable 4: Birth weight of neonates receiving PN in the first postnatal week as proportion of live births

Confidential: For Review Only

eTable 1: Data fields for extraction from NNRD

**Outcome**	
**Variable**	**Data items**
Parental nutrition	**PN group** defined as<br>Any of the following items entered in the 'Daily Care Fluids' and 'Feeding' during first 7 days:<br>• Y entry for PARENTERAL NUTRITION RECEIVED INDICATOR<br>Or<br>The following drug code entered in the Daily care medication during first 7 days:<br>• 1010238 Total parenteral nutrition<br><br>**No PN group** defined as<br>All other babies

**Variables**	
**Variable**	**Data items**
Gestational age at birth	Data extracted from GESTATION LENGTH (AT DELIVERY):
Birth weight	Data extracted from BIRTH WEIGHT
Neonatal network	Data extracted from SITE CODE (OF ACTUAL PLACE OF DELIVERY)

Confidential: For Review Only

eTable 2: Gestational age of neonates receiving PN in the first postnatal week as proportion of neonatal unit admissions

Gestational age category† at birth	Neonates receiving PN in the first postnatal week by year of birth					
	2012	2013	2014	2015	2016	2017
Extremely preterm	2317 *(92)*	2309 *(94)*	2267 *(94)*	2348 *(94)*	2421 *(96)*	2325 *(95)*
Very preterm	3493 *(65)*	3900 *(72)*	3896 *(73)*	4059 *(74)*	4135 *(75)*	4110 *(75)*
Moderate and late preterm	2343 *(9.3)*	2640 *(10)*	2796 *(11)*	2683 *(9.7)*	2547 *(9.2)*	2375 *(8.4)*
Term	1370 *(2.9)*	1688 *(3.4)*	1618 *(3.0)*	1528 *(2.7)*	1484 *(2.5)*	1493 *(2.3)*

†Gestational age at birth categorised using WHO definitions (33)
Extremely preterm: <28^{+0} weeks, Very preterm: 28^{+0} - 31^{+6} weeks, Moderate and late preterm; 32^{+0} - 36^{+6} weeks, Term >36^{+6} weeks
Number in brackets indicates the percentage of all live births given PN in each category (Denominator data from NNRD)

Confidential: For Review Only

eTable 3: Proportion of live births receiving PN in the first postnatal week by year of birth

Gestational age in weeks at birth	Neonates receiving PN in the first postnatal week by year of birth (%)						Trend analysis p value
	2012	2013	2014	2015	2016	2017	
<22	0.4	1.7	1.3	0.7	1.8	2.3	0.12
23	51	61	58	63	66	71.2	0.01
24	79	80	86	91	89	86	0.12
25	88	93	97	91	97	94	0.28
26	88	91	96	94	92	95	0.16
27	91	94	93	98	99	97	0.03
28	86	94	94	95	98	96	0.06
29	81	89	91	91	93	93	0.04
30	56	69	71	71	73	74	0.04
31	41	50	50	53	51	52	0.08
32	24	30	31	31	31	31	0.11
33	14	16	17	16	15	13	0.63
34	6.8	8.1	8.4	7.2	7.6	6.3	0.50
35	3.0	3.3	3.8	3.3	3.2	3.1	0.91
36	1.6	1.7	1.7	1.6	1.4	1.3	0.09
37	0.7	0.8	0.7	0.7	0.7	0.7	0.28
38	0.3	0.4	0.4	0.3	0.3	0.3	0.82
39	0.1	0.2	0.2	0.2	0.2	0.2	0.76
40	0.1	0.2	0.2	0.2	0.2	0.2	0.75
41	0.2	0.2	0.2	0.2	0.2	0.2	0.32
42	0.1	0.2	0.2	0.2	0.2	0.2	0.42

Denominator data from total number of live births from ONS Birth characteristics in England and Wales
Trend analysis for proportion of live births given PN in each category was completed: none of the trends over time were significant after Bonferroni-Holm correction for multiple comparisons
Neonates with missing data for gestational age = 3

Confidential: For Review Only

eTable 4: Birth weight of neonates receiving PN in the first postnatal week as proportion of live births

Birth weight category* at birth	Neonates receiving PN in the first postnatal week by year of birth						Trend analysis p value
	2012	2013	2014	2015	2016	2017	
<1kg	2227 *(67)*	2578 *(72)*	2594 *(73)*	2792 *(80)*	2724 *(82)*	2533 *(100)*†	0.01
<1.5kg	5375 *(70)*	5935 *(78)*	6054 *(81)*	6341 *(84)*	6393 *(88)*	6087 *(96)*	0.00
<2.5kg	7727 *(16)*	8359 *(18)*	8444 *(18)*	8883 *(18)*	8939 *(18)*	8629 *(18)*	0.06
>2.5kg	1290 (0.2)	1550 (0.3)	1504 (0.3)	1739 (0.3)	1655 (0.3)	1674 (0.3)	0.60

**Birth weight categorised using WHO definitions (34)*
Number in brackets indicates the percentage of all live births given PN in each category (Denominator data from ONS Birth characteristics in England and Wales)
Trend analysis for proportion of neonates admitted to a neonatal unit given PN in each category: p value was significant after Bonferroni-Holm correction for multiple comparisons for <1kg and <1.5kg categories
Neonates with missing data for birth weight = 4
†The ONS changed the way they clean birthweight data in 2017 and as a result the number of neonates born <1kg dropped from 3117 births in 2016 to 2538 in 2017 This change in the denominator inflated the proportion of <1kg births given PN in 2017.

Confidential: For Review Only

BMJ Paediatrics Open

Use of parenteral nutrition in the first postnatal week in England and Wales: An observational study using real-world data

Journal:	*BMJ Paediatrics Open*
Manuscript ID	bmjpo-2022-001543.R1
Article Type:	Original research
Date Submitted by the Author:	08-Jun-2022
Complete List of Authors:	Webbe, James; Imperial College London, Academic Neonatal Medicine<br>Battersby, Cheryl; Imperial College London, Medicine<br>Longford, Nicholas ; Imperial College London, Department of Medicine<br>Oughham , Kayleigh; Imperial College London Faculty of Medicine, Neonatal Data Analysis Unit<br>uthaya, s; Imperial College London, Neonatal Unit<br>Modi, Neena; Imperial College London, Neonatal Medicine<br>Gale, Chris; Imperial College London, Academic Neonatal Medicine
Keywords:	Neonatology, Epidemiology

SCHOLARONE™
Manuscripts

I, the Submitting Author has the right to grant and does grant on behalf of all authors of the Work (as defined in the below author licence), an exclusive licence and/or a non-exclusive licence for contributions from authors who are: i) UK Crown employees; ii) where BMJ has agreed a CC-BY licence shall apply, and/or iii) in accordance with the terms applicable for US Federal Government officers or employees acting as part of their official duties; on a worldwide, perpetual, irrevocable, royalty-free basis to BMJ Publishing Group Ltd ("BMJ") its licensees and where the relevant Journal is co-owned by BMJ to the co-owners of the Journal, to publish the Work in this journal and any other BMJ products and to exploit all rights, as set out in our licence.

The Submitting Author accepts and understands that any supply made under these terms is made by BMJ to the Submitting Author unless you are acting as an employee on behalf of your employer or a postgraduate student of an affiliated institution which is paying any applicable article publishing charge ("APC") for Open Access articles. Where the Submitting Author wishes to make the Work available on an Open Access basis (and intends to pay the relevant APC), the terms of reuse of such Open Access shall be governed by a Creative Commons licence – details of these licences and which Creative Commons licence will apply to this Work are set out in our licence referred to above.

Other than as permitted in any relevant BMJ Author's Self Archiving Policies, I confirm this Work has not been accepted for publication elsewhere, is not being considered for publication elsewhere and does not duplicate material already published. I confirm all authors consent to publication of this Work and authorise the granting of this licence.

Confidential: For Review Only

Use of parenteral nutrition in the first postnatal week in England and Wales: An observational study using real-world data

J Webbe[1], C Battersby[2], N Longford[3], K Ougham[4], S Uthaya[5], N Modi[6], C Gale[7]

[1] James Webbe, PhD

Neonatal Medicine, School of Public Health, Faculty of Medicine, Imperial College London

j.webbe@imperial.ac.uk

[2] Cheryl Battersby, PhD

Neonatal Medicine, School of Public Health, Faculty of Medicine, Imperial College London

c.battersby@imperial.ac.uk

[3] Nicholas Longford, PhD

Neonatal Medicine, School of Public Health, Faculty of Medicine, Imperial College London

n.longford@imperial.ac.uk

[4] Kayleigh Ougham, MSc

Neonatal Medicine, School of Public Health, Faculty of Medicine, Imperial College London

k.ougham@imperial.ac.uk

[5] Sabita Uthaya, MD

Neonatal Medicine, School of Public Health, Faculty of Medicine, Imperial College London

s.uthaya@imperial.ac.uk

[6] Neena Modi, FMedSci

Confidential: For Review Only

Neonatal Medicine, School of Public Health, Faculty of Medicine, Imperial College London

n.modi@imperial.ac.uk

[7] Chris Gale, PhD

Neonatal Medicine, School of Public Health, Faculty of Medicine, Imperial College London

christopher.gale@imperial.ac.uk

Corresponding author

Dr Christopher Gale, Reader in Neonatal Medicine

Neonatal Medicine, Chelsea and Westminster campus, Imperial College London

369 Fulham Road, London, SW10 9NX, United Kingdom.

+ 44 (0)203 3155418

christopher.gale@imperial.ac.uk

Manuscript word count: 2252

Confidential: For Review Only

ABSTRACT

Background

Parenteral nutrition (PN) is used to provide supplemental support to neonates while enteral feeding is being established. PN is a high-cost intervention with beneficial and harmful effects. Internationally there is substantial variation in how PN is used, and there are limited contemporary data describing use across the UK.

Objective

To describe PN use in the first postnatal week in infants born and admitted to neonatal care in England, Scotland and Wales.

Method

Data describing neonates admitted to National Health Service (NHS) neonatal units between 1st January 2012 and 31st December 2017, extracted from routinely recorded data held the National Neonatal Research Database (NNRD); the denominator was live births, from Office for National Statistics.

Results

Over the study period 62,145 neonates were given PN in the first postnatal week (1.4% of all live births); use was higher in more preterm neonates (76% of livebirths at <28 weeks, 0.2% of term livebirths) and in neonates with lower birth weight. 15% (9181/62145) of neonates given PN in the first postnatal week were born at term. There was geographic variation in PN administration: the proportion of live births given PN within neonatal regional networks ranged from 1.0% (95% confidence intervals: 1.0, 1.0) to 2.8% (95% confidence interval: 2.7, 2.9).

Conclusions and relevance

Significant variation exists in neonatal PN use; it is unlikely this reflects optimal use of an expensive intervention. Research is needed to identify which babies will benefit most and which are at risk of harm from early PN.

Registration

ClinicalTrials.gov: NCT03767634

https://mc.manuscriptcentral.com/bmjpo

Confidential: For Review Only

Registration date: 06/12/2018

Abstract word count: 250/250

Confidential: For Review Only

INTRODUCTION

In 1968 parenteral nutrition (PN) was used to support the metabolic needs of a term neonate with small bowel atresia (1). Following this, PN has been increasingly used to supplement the nutrition of sick or preterm neonates. The widespread use of PN has been encouraged on the basis that optimising nutrition will improve short and long-term outcomes (2). It is considered most beneficial for neonates born preterm or with lower birth weight who have fewer reserves and may accrue large nutritional deficits before enteral feeds are established (2). Despite widespread use, the impact of PN on key neonatal outcomes has not been evaluated in randomised controlled neonatal trials powered for clinically meaningful and functional end-points.

Therefore, while effects on short-term biochemical markers such as nitrogen balance are well described (3), evidence to support beneficial effects on survival and neurodevelopment are lacking (3, 4). Conversely, PN carries well described risks, of which the most serious and common is bloodstream infection (5). Recent evidence from large randomised controlled trials in critically unwell adults (6) and children (7) showed that use of PN during the first seven days of admission to an intensive care unit led to worse outcomes, when compared to delayed PN administration, indicating that the harms of early PN outweigh benefits in these populations. Although there has not been a similar trial in neonatal care, subgroup analysis in the PePaNIC trial of term neonates looked after on paediatric intensive care units also showed increased rates of nosocomial infection with early PN use (8), suggesting that early PN use should be targeted at neonates with most potential for benefit.

Given the uncertain balance of risk and benefit for neonatal PN use, it is unsurprising that international practice is variable: some neonatal units in high-income countries provide PN to up to 70% of neonatal admissions (9) while others report not using PN (10). In the United Kingdom, a 2011 report from the National Confidential Enquiry into Patient Outcome and Death found considerable variation in neonatal management in 2008 with only 24% of patients receiving PN that was considered best practice (11). Following this, a national

Confidential: For Review Only

framework (12), and NICE guidance (13) have been developed. The most recent NICE guidance makes recommendations about prescription, administration, monitoring and recipients of PN in neonatal units; recommending all neonates born before 30^{+0} weeks^{+days} gestation or weighing under 1250g at birth, and any who are unable (or not expected) to establish milk feeds of ≥100mL/kg/day by postnatal day five, receive early PN.

We aimed to describe how PN is used in the first postnatal week, to explore how use is influenced by gestational age, birth weight, geographical region, and to compare how use has changed over time in the period prior to the publication of 2020 NICE guidance.

OBJECTIVE

To describe the pattern of PN use in neonatal units in England, Scotland and Wales in the first seven postnatal days.

METHODS

Study design

This study was an epidemiological description of practice: we pre-registered it (Clinicaltrials.gov: NCT03767634) and published the study protocol (14). We report it in line with RECORD guidelines (15).

Data source

We used de-identified data held in the National Neonatal Research Database (NNRD) (16). The NNRD holds data extracted from electronic health records completed by health professionals during routine clinical care (17). The Neonatal Data Set, a defined national data standard (18) comprising approximately 450 items, is extracted and transmitted to the Neonatal Data Analysis Unit at Imperial College London. The NNRD holds data from all neonates admitted to NHS neonatal units in England, Scotland and Wales; in total the NNRD contains data from about one million neonates from 2008 to the present. Accuracy and

Confidential: For Review Only

completeness of NNRD data have been confirmed by comparison with Case Record Forms from a prospective clinical trial, which showed high data completeness and accuracy (>95%) (19). The Chief Investigator of the NNRD (NM) holds UK Research Ethics Committee approval (REC Reference: 16/LO/1093), and Confidentiality Advisory Group approval (ECC 8-05(f/2010)) for the NNRD as a research database. Study specific Research Ethics Committee (18/NI/0214), Health Research Authority and Health and Care Research Wales approval and approval for inclusion of their data in this study from all English neonatal units was obtained. Data for this study was extracted by author KO, operating within the guidelines established by these approvals; no other investigators had accessed the wider NNRD for this study. No data cleaning methods were required for this study, and no data linkage was required. We obtained population-level data for total live births by gestational age and birth weight from the Office for National Statistics (ONS) (20-25), and for live births by neonatal network from Mothers and Babies: Reducing Risk through Audits and Confidential Enquiries across the UK (MBRRACE-UK) reports (26-29), for denominator data. Population-level data for total neonatal unit admissions by gestational age and birth weight were obtained from the NNRD for denominator data.

Confidential: For Review Only

Participants

The study population was all neonates born between 1st January 2012 and 31st December 2017 and admitted to a neonatal unit in England and Wales. There were no exclusion criteria: this was intended to maximise the sample size and ensure complete, population-level data.

Variables

The primary outcome was any use of PN in the first seven postnatal days. To describe the background characteristics of neonates given PN we extracted data relating to gestational age at birth, birth weight, year of birth, and neonatal network (30) of birth. The variables extracted from the NNRD, and how they were defined, are listed in eTable 1.

Statistical methods

We described the characteristics of neonates that received PN in the first seven postnatal days and compared use between different groups. For gestational age, we grouped neonates by completed weeks of gestation according to WHO categories (31). For birth weight, we grouped neonates according to WHO categories (32). To explore differences in PN use across geographical regions, we grouped neonates by neonatal network of birth (30). We compared changes in PN prescribing over time by grouping neonates according to the year of birth.

We used the Student's t-test to test differences in rates of PN among different groups, and a linear regression t-test to test for a change in PN use within different categories over time. We corrected for multiple comparisons using the Bonferroni-Holm method (33, 34).

RESULTS

The only deviation from the protocol related to the use of data from neonatal units in Scotland. We were unable to use data on babies from Scottish neonatal units due to difficulties obtaining the institutional approvals required for Public Benefit and Privacy Panel for Health and Social Care (35) approval. The project was completed using data from neonatal units in England and Wales only.

Over the six-year study period 4,196,314 neonates were born in England and Wales. Of these, 347,959 neonates were admitted to NHS neonatal units and had data held within the NNRD; 62,145 were recorded as having received PN during the first postnatal week. We did not identify a significant difference over time in the proportion of neonates that received PN: just over 1% of all live births (Table 1) and 17% of all neonatal unit admissions (eTable 2). PN was given to neonates of all gestational ages, with neonates born preterm more likely to be recipients. However, a large proportion of the neonates who receive PN were more mature: 15% of neonates who received PN in the first postnatal week over the study period were born at term. We did not find a significant change in PN use over this period in any gestational age category after correction for multiple comparisons (Table 1).

Confidential: For Review Only

Table 1 Gestational age of neonates receiving PN in the first postnatal week as a proportion of total live births

Gestational age category* at birth	Neonates receiving PN in the first postnatal week by year of birth n (% of live births)						Trend analysis p value
	2012	2013	2014	2015	2016	2017	
Extremely preterm	2317 *(72)*	2309 *(75)*	2267 *(77)*	2348 *(78)*	2421 *(76)*	2325 *(75)*	0.14
Very preterm	3493 *(61)*	3900 *(71)*	3896 *(71)*	4059 *(73)*	4135 *(73)*	4110 *(74)*	0.03
Moderate and late preterm	2343 *(5.3)*	2640 *(6.1)*	2796 *(6.4)*	2683 *(6.0)*	2547 *(5.6)*	2375 *(5.2)*	0.52
Term	1370 *(0.2)*	1688 *(0.3)*	1618 *(0.3)*	1528 *(0.2)*	1484 *(0.2)*	1493 *(0.2)*	0.04
Total	9523 *(1.3)*	10537 *(1.5)*	10577 *(1.5)*	10618 *(1.5)*	10587 *(1.5)*	10303 *(1.5)*	0.02

**Gestational age at birth categorised using WHO definitions (31)*
Extremely preterm: <28^{+0} weeks, Very preterm: 28^{+0} - 31^{+6} weeks, Moderate and late preterm; 32^{+0} - 36^{+6} weeks, Term >36^{+6} weeks
Number in brackets indicates the percentage of all live births given PN in each category (Denominator data from ONS Birth characteristics in England and Wales)
Trend analysis for proportion of live births given PN in each category: none of the trends over time were significant after Bonferroni-Holm correction for multiple comparisons
Neonates with missing data for gestational age = 3

The proportion of live births who receive PN in the first postnatal week is lower in neonates born at 22 or 23 weeks, rises to over 90% for neonates born from 25 to 28 weeks gestation before falling again (Figure 1). When analysed by gestational age no change over the six years was found in PN use in the first postnatal week; no significant trend was observed after correction for multiple comparisons (eTable 3).

Neonates born with lower birth weights were more likely to receive PN in the first postnatal week (Table 2, eTable 4). A significant increase in PN administration was seen among neonates with birthweight <1.5kg over the study period.

Table 2 Birth weight of neonates receiving PN in the first postnatal week as proportion of neonatal unit admissions

Confidential: For Review Only

Birth weight at birth*	Neonates receiving PN in the first postnatal week by year of birth<br>n (% of neonatal units admissions)						Trend analysis p value
	2012	2013	2014	2015	2016	2017	
<1kg	2401 *(92)*	2484 *(94)*	2442 *(95)*	2542 *(95)*	2547 *(96)*	2541 *(96)*	0.001
<1.5kg	5591 *(77)*	5878 *(82)*	5936 *(83)*	6090 *(84)*	6213 *(85)*	6095 *(85)*	0.01
<2.5kg	7999 *(27)*	8679 *(29)*	8756 *(29)*	8880 *(28)*	8935 *(28)*	8628 *(28)*	0.16
>2.5kg	1524 *(3.1)*	1857 *(3.5)*	1821 *(3.2)*	1738 *(2.8)*	1652 *(2.6)*	1675 *(2.4)*	0.3

**Birth weight categorised using WHO definitions (32)*
Number in brackets indicates the percentage of all neonates admitted to a neonatal unit given PN in each category (Denominator data from NNRD)
Trend analysis for proportion of neonates admitted to a neonatal unit given PN in each category: p value was significant after Bonferroni-Holm correction for multiple comparisons for <1kg and <1.5kg categories
Neonates with missing data for birth weight = 4

The use of PN differed across networks, with a range from 1.0% to 2.8% of all live births given PN in the first postnatal week (Figure 2); these were significantly different from the national average, 1.5% all live births, for nine of the 14 neonatal networks (Table 4). Rates of PN administration within neonatal networks varied less over time than the differences seen between networks.

Table 3 Neonates receiving PN in the first postnatal week by Neonatal Network as proportion of live births

Neonatal Network at birth	Babies receiving PN in the first postnatal week by year of birth<br>n (% of live births)					
	2012	2013	2014	2015	2016	2017
East Midlands[1]	608 *(†)*	601 *(1.1%)*	607 *(1.1%)*	553 *(1.0%)*	570 *(1.0%)*	566 *(1.0%)*
East of England	953 *(†)*	973 *(1.4%)*	1052 *(1.5%)*	942 *(1.4%)*	961 *(1.4%)*	926 *(1.4%)*
London - North Central & East	771 *(†)*	733 *(1.4%)*	806 *(1.5%)*	842 *(1.6%)*	845 *(1.6%)*	845 *(1.6%)*
London - North West	417 *(†)*	429 *(1.4%)*	421 *(1.3%)*	403 *(1.3%)*	443 *(1.4%)*	409 *(1.3%)*
London - South	725 *(†)*	723 *(1.6%)*	739 *(1.7%)*	804 *(1.8%)*	828 *(1.8%)*	764 *(1.7%)*
North West	1233 *(†)*	1206 *(1.5%)*	1222 *(1.5%)*	1231 *(1.5%)*	1130 *(1.3%)*	1031 *(1.2%)*
Northern	326 *(†)*	398 *(1.2%)*	397 *(1.2%)*	408 *(1.3%)*	413 *(1.3%)*	363 *(1.1%)*

Confidential: For Review Only

South East Coast	681 (†)	723 *(1.5%)*	707 *(1.5%)*	720 *(1.5%)*	717 *(1.5%)*	710 *(1.5%)*
South West	849 (†)	805 *(1.7%)*	785 *(1.6%)*	839 *(1.8%)*	813 *(1.7%)*	786 *(1.6%)*
Southern West Midlands	425 (†)	426 *(1.3%)*	443 *(1.4%)*	431 *(1.4%)*	484 *(1.5%)*	454 *(1.5%)*
Staffordshire, Shropshire, & Black Country	495 (†)	534 *(2.1%)*	527 *(2.1%)*	553 *(2.3%)*	479 *(1.9%)*	499 *(2.0%)*
Thames Valley & Wessex	1062 (†)	1068 *(1.9%)*	1021 *(1.7%)*	1052 *(1.8%)*	1045 *(1.7%)*	1034 *(1.8%)*
Wales	166 (†)	933 *(2.9%)*	910 *(2.9%)*	881 *(2.8%)*	846 *(2.7%)*	888 *(2.8%)*
Yorkshire & Humber	692 (†)	820 *(1.2%)*	791 *(1.2%)*	795 *(1.2%)*	847 *(1.3%)*	878 *(1.3%)*

[1]East Midlands Operational Delivery Network previously the separate Central and Trent Networks
Number in brackets indicates the percentage of all live births given PN in each Neonatal Network (Data from MBRRACE-UK reports)
†MBRRACE-UK report not produced for 2012
Neonates with missing data for neonatal network = 202

DISCUSSION

In this work, we described the characteristics of neonates born in England and Wales who receive PN in the first postnatal week. PN is a common intervention and is given to 17% of all neonatal unit admissions. While PN use is higher in neonates born prematurely and with lower birth weight, a considerable proportion are born at term. We also show that use varies between different neonatal networks.

That higher rates of PN use is seen in neonates born more preterm is unsurprising, as these populations are considered most likely to benefit. The lower rates seen in the most preterm neonates (those born at <26 gestational weeks) are likely because many of these babies die before admission to a neonatal unit and before PN is commenced. However, because of the much larger proportion of babies born at more mature gestations it is noteworthy that around 15% of all babies given PN are born at term. The energy requirements (36), indications for use and metabolic stability of these groups differs, thus the risks and benefits of PN may also differ substantially across gestational ages.

Confidential: For Review Only

We found PN use in the first postnatal week varied significantly between neonatal networks. This is in keeping with variations between neonatal units in PN use in other settings (9, 10). However, we were unable to find another comprehensive population based study of national PN use. From our descriptive analysis, it is not possible to determine whether the variation we identified is explained by regional differences in rates of prematurity, neonatal sickness or other case-mix factors (such as congenital anomalies or neonatal surgical conditions), nor, given the paucity of evidence, is it possible to comment on what rate of PN use should be expected. However, by identifying wide variation in practice within the United Kingdom, we highlight early PN use as an area where optimal practice is uncertain (37). This variation also has financial implications. Treating a neonate with PN for one day in 2012 cost £37.43 (38), and if all hospitals in England and Wales treated the same proportion of live births as the highest use network this would cost the NHS an additional £2.5 million each year. This expense may be justified if PN leads to lower mortality or morbidity, but such evidence is lacking.

The strengths of our study include the population-level coverage involving a cohort of over 4 million neonates. The population-level data meant that recruitment bias was reduced, but not fully eliminated. The NNRD covers all neonatal units in England and Wales but does not include Paediatric Intensive Care Units and surgical units that admit neonates. Some term cardiac and surgical babies will be cared for on these units and thus we may have underestimated the amount of PN used in these groups. We followed a pre-specified protocol and data analysis plan and limited the risk of false discovery associated with multiple comparisons (39) by using the Holm-Bonferroni method. In keeping with previous studies that have used the NNRD, we had very little missing data (19, 40). Limitations of this study include that we were not able to obtain permission to use data for neonates born in Scotland, reducing the study population. As the NNRD only holds data for neonates admitted to an NHS neonatal unit for denominator data, we used data from the ONS and MBRRACE-UK to provide total numbers of live births. As MBRRACE-UK did not produce a report in 2012 this limited the

Confidential: For Review Only

number of years for which we could undertake network level comparisons. As has been found in previous studies, due to the lack of additional information about PN in the data extracted we are unable to describe changes in how PN was used (e.g. when PN was commenced or types of PN used) (41-43).

Our findings show that PN use in the first postnatal week is common in England and Wales, with regional variation. In light of the potential harm found with early PN in critically unwell children (7), and the lack of evidence of benefit for clinically important endpoints in infants (3, 44) research is needed to ensure that PN use in neonates is underpinned by a robust evidence base. Our data will help in the planning of future trials to identify which neonates will benefit from early PN and ensure that clinical practice is based on strong evidence. Approximately twenty-eight neonates are started on PN each day in England and Wales: if all of these diverse patients are to receive optimal treatment urgent research is needed to ensure they are the neonates who will benefit most from this intervention, and to avoid harm in others.

CONCLUSION

Parenteral nutrition is commonly used in the first postnatal week across the United Kingdom, with higher use in neonates born more preterm. Across all gestational age categories no change in PN use in the first postnatal week over time was found, but there is persisting variation in use between regional neonatal networks. Research is needed to ensure PN use in this group is well targeted.

Confidential: For Review Only

ABBREVIATIONS

ELBW	Extremely Low Birth Weight
LBW	Low Birth Weight
MBRRACE-UK	Mothers and Babies: Reducing Risk through Audits and Confidential Enquiries across the UK
NNRD	National Neonatal Research Database
ONS	Office for National Statistics
PICU	Paediatric Intensive Care Unit
PN	Parenteral Nutrition
VLBW	Very Low Birth Weight

Confidential: For Review Only

DECLARATIONS

Consent for publication

Not applicable

Availability of data and material

Applications to use the data used within this project should be made to the Neonatal Data Analysis Unit, Imperial College London

Competing interests

JW has received support from Chiesi Pharmaceuticals to attend an educational conference and has received a research grant from Mason Medical Research Foundation.

SU has received funding from the National Institute of Health Research, the Department of Health and Prolacta Life Sciences. SU has been on the Advisory Board of Fresenius Kabi and received honoraria and travel expenses for speaking at study days organised by Fresenius Kabi. SU is a member of the National Institute for Health and Care Excellence Parenteral Nutrition Guideline Development Committee. NM is the Chief Investgator for the National Neonatal Research Database. In the last five years NM has served on the Board of Trustees of the Royal College of Paediatrics and Child Health, David Harvey Trust, Medical Women's Federation, Medact, Action Cerebral Palsy, TheirWorld and Academy of Medical Sciences. NM is a member of the Nestle Scientific Advisory Board but accepts no personal remuneration for this role. NM has received research grants from the British Heart Foundation, Medical Research Council, National Institute of Health Research, Westminster Research Fund, Prolacta Life Sciences, Chiesi, Takeda, March of Dimes, Health Data Research UK, HCA International, and European Health Data Evidence Network; travel and accommodation expenses from Prolacta, Nestle and Chiesi.

Confidential: For Review Only

CG has received support from Chiesi Pharmaceuticals to attend an educational conference; in the past 5 years he been investigator on received research grants from Medical Research Council, National Institute of Health Research, Canadian Institute of Health Research, Department of Health in England, Mason Medical Research Foundation, Westminster Medical School Research Trust and Chiesi Pharmaceuticals.

CB has received support from Chiesi Pharmaceuticals to attend an educational conference; she is supported by a personal award by the National Institute of Health Research.

Funding

The NNRD is created and maintained through grants and unrestricted funding held by NM. This includes costs involved in data transfer, storage, cleaning, merging, administration and regulatory approvals. The extraction of study data from the NNRD and analysis for this study was funded through a Mason Medical Research Fellowship awarded to JW. CG is funded by the United Kingdom Medical Research Council (MRC) through a Clinician Scientist Fellowship award. The MRC and Mason Medical Research Foundation were not involved in the design of the study, collection, analysis, and interpretation of data or in writing the manuscript

Authors' contributions

JW, CG and NM conceived this project. JW, CG, KO and NL planned the statistical analyses. The first draft of the manuscript was written by JW and revised by NM; CG, SU, CB, NL and NM edited and reviewed the manuscript. It was approved by JW, CG, CB, KO, NL, SU and NM.

Acknowledgements

We wish to thank Angela Richard-Löndt and Laura Noakes, parents of preterm infants, for their support in developing this research project and BLISS for their input and support developing the dissemination plan.

Confidential: For Review Only

What is already known on this topic?

- Parenteral nutrition is commonly given to neonates unable to receive adequate milk feeds or while milk feeds are introduced
- There is limited evidence to inform parenteral nutrition use in neonates

What this study adds?

- The early use of parenteral nutrition is common; 17% of all babies admitted to neonatal units receive PN in the first postnatal week
- Fifteen percent of neonates given PN in the first postnatal week are born full-term
- There is variation in the use of parenteral nutrition in the first week of postnatal life between regional neonatal networks

Confidential: For Review Only

REFERENCES

1. Wilmore DW, Dudrick SJ. Growth and Development of an Infant Receiving All Nutrients Exclusively by Vein. Jama. 1968;203(10):860-4.
2. Ziegler EE, Thureen PJ, Carlson SJ. Aggressive nutrition of the very low birthweight infant. Clinics in perinatology. 2002;29(2):225-44.
3. Trivedi A, Sinn JK. Early versus late administration of amino acids in preterm infants receiving parenteral nutrition. Cochrane Database Syst Rev. 2013(7):CD008771.
4. Moyses HE, Johnson MJ, Leaf AA, Cornelius VR. Early parenteral nutrition and growth outcomes in preterm infants: a systematic review and meta-analysis. The American journal of clinical nutrition. 2013;97(4):816-26.
5. Zingg W, Tomaske M, Martin M. Risk of parenteral nutrition in neonates--an overview. Nutrients. 2012;4(10):1490-503.
6. Casaer MP, Mesotten D, Hermans G, Wouters PJ, Schetz M, Meyfroidt G, et al. Early versus late parenteral nutrition in critically ill adults. N Engl J Med. 2011;365(6):506-17.
7. Fivez T, Kerklaan D, Mesotten D, Verbruggen S, Wouters PJ, Vanhorebeek I, et al. Early versus Late Parenteral Nutrition in Critically Ill Children. N Engl J Med. 2016;374(12):1111-22.
8. van Puffelen E, Vanhorebeek I, Joosten KFM, Wouters PJ, Van den Berghe G, Verbruggen S. Early versus late parenteral nutrition in critically ill, term neonates: a preplanned secondary subgroup analysis of the PEPaNIC multicentre, randomised controlled trial. The Lancet Child & adolescent health. 2018;2(7):505-15.
9. Christensen RD, Henry E, Wiedmeier SE, Burnett J, Lambert DK. Identifying patients, on the first day of life, at high-risk of developing parenteral nutrition-associated liver disease. Journal of perinatology : official journal of the California Perinatal Association. 2007;27(5):284-90.
10. Ksiazyk J, Swietlinski J, Brozek G, Musialik-Swietlinska E, Migdal M, Zejda JE. [Parenteral nutrition in neonatal units: a survey of current practices in Poland]. Medycyna wieku rozwojowego. 2008;12(4 Pt 1):899-904.
11. Mason DG, Puntis JWL, McCormick K, Smith N. Parenteral nutrition for neonates and children: a mixed bag. Archives of Disease in Childhood. 2011;96(3):209.
12. British Association of Perinatal Medicine. The Provision of Parenteral Nutrition within Neonatal Services - A Framework for Practice [Guideline]. 2016 [updated April 2016. Available from: http://www.bapm.org/publications/documents/guidelines/Parenteral%20Nutrition%20April%202016.pdf.
13. Excellence; NIfHaC. Neonatal parenteral nutrition: NICE guideline [NG154]. 2020.
14. Webbe J, Longford N, Uthaya S, Modi N, Gale C. Outcomes following early parenteral nutrition use in preterm neonates: protocol for an observational study. BMJ open. 2019;9(7):e029065.
15. Benchimol EI, Smeeth L, Guttmann A, Harron K, Moher D, Petersen I, et al. The REporting of studies Conducted using Observational Routinely-collected health Data (RECORD) Statement. PLOS Medicine. 2015;12(10):e1001885.
16. Gale C, Morris I. The UK National Neonatal Research Database: using neonatal data for research, quality improvement and more. Archives of disease in childhood Education and practice edition. 2016;101(4):216-8.
17. Spencer A, Modi N. National neonatal data to support specialist care and improve infant outcomes. Arch Dis Child Fetal Neonatal Ed. 2013;98(2):F175-80.
18. NHS Digital. National Neonatal Data Set 2017 [Available from: http://www.datadictionary.nhs.uk/data_dictionary/messages/clinical_data_sets/data_sets/national_neonatal_data_set/national_neonatal_data_set_-_episodic_and_daily_care_fr.asp?shownav=1.

Confidential: For Review Only

19. Battersby C, Statnikov Y, Santhakumaran S, Gray D, Modi N, Costeloe K, et al. The United Kingdom National Neonatal Research Database: A validation study. PloS one. 2018;13(8).
20. Office for National Statistics. Characteristics of Birth 1, England and Wales: 2012. Fareham: Vital Statistics Outputs Branch, Office for National Statistics; 2013.
21. Office for National Statistics. Characteristics of Birth 1, England and Wales: 2013. Fareham: Vital Statistics Outputs Branch, Office for National Statistics; 2014.
22. Office for National Statistics. Birth Characteristics in England and Wales: 2014. Fareham: Vital Statistics Outputs Branch, Office for National Statistics; 2015.
23. Office for National Statistics. Birth Characteristics in England and Wales: 2015. Fareham: Vital Statistics Outputs Branch, Office for National Statistics; 2016.
24. Office for National Statistics. Birth Characteristics in England and Wales: 2016. Fareham: Vital Statistics Outputs Branch, Office for National Statistics; 2017.
25. Office for National Statistics. Birth Characteristics, England and Wales: 2017. Fareham: Vital Statistics Outputs Branch, Office for National Statistics; 2018.
26. Draper ES, Gallimore ID, Kurinczuk JJ, Smith PW, Boby T, Smith LK, et al. MBRRACE-UK Perinatal Mortality Surveillance Report, UK Perinatal Deaths for Births from January to December 2016. Leicester: The Infant Mortality and Morbidity Studies, Department of Health Sciences, University of Leicester; 2018.
27. Manktelow BN, Smith LK, Prunet C, Smith PW, Boby T, Hyman-Taylor P, et al. MBRRACE-UK Perinatal Surveillance Report: UK Perinatal Deaths for Births from January to December 2015. Leicester: The Infant Mortality and Morbidity Studies, Department of Health Sciences, University of Leicester; 2017.
28. Manktelow BN, Smith LK, Seaton SE, Hyman-Taylor P, Kurinczuk JJ, Field DJ, et al. MBRRACE-UK Perinatal Mortality Surveillance Report, UK Perinatal Deaths for Births from January to December 2014. Leicester: The Infant Mortality and Morbidity Studies, Department of Health Sciences, University of Leicester; 2016.
29. Manktelow BN, Smith LK, Evans TA, Hyman-Taylor P, Kurinczuk JJ, Field DJ, et al. Perinatal Mortality Surveillance Report UK Perinatal Deaths for births from January to December 2013. Leicester: The Infant Mortality and Morbidity Group, Department of Health Sciences, University of Leicester; 2015.
30. Gale C, Santhakumaran S, Nagarajan S, Statnikov Y, Modi N, Unit NDA, et al. Impact of managed clinical networks on NHS specialist neonatal services in England: population based study. Brit Med J. 2012;344.
31. Blencowe H, Cousens S, Chou D, Oestergaard M, Say L, Moller AB, et al. Born Too Soon: The global epidemiology of 15 million preterm births. Reprod Health. 2013;10.
32. World Health Organization. International Statistical Classification of Diseases and Related Health Problems 10th Revision. 2016.
33. Proschan MA, Waclawiw MA. Practical Guidelines for Multiplicity Adjustment in Clinical Trials. Control Clin Trials. 2000;21(6):527-39.
34. Holm S. A Simple Sequentially Rejective Multiple Test Procedure. Scand J Stat. 1979;6(2):65-70.
35. Morris C, Gray A, Scott J, editors. NHS Scotland Public Benefit and Privacy Panel (PBPP) – Much Ado about Governance 3 years on. International Population Data Linkage Conference; 2018; Banff, Canada.
36. Schanler RJ, Garza C, Nichols BL. Fortified mothers' milk for very low birth weight infants: results of growth and nutrient balance studies. J Pediatr. 1985;107(3):437-45.
37. Soll RF, McGuire W. Evidence-Based Practice: Improving the Quality of Perinatal Care. Neonatology. 2019:1-6.
38. Walter E, Liu FX, Maton P, Storme T, Perrinet M, von Delft O, et al. Cost analysis of neonatal and pediatric parenteral nutrition in Europe: a multi-country study. European Journal Of Clinical Nutrition. 2012;66:639.
39. Young SS, Karr A. Deming, data and observational studies. Significance. 2011;8(3):116-20.

https://mc.manuscriptcentral.com/bmjpo

Confidential: For Review Only

40. Battersby C, Longford N, Mandalia S, Costeloe K, Modi N, group UKNCNEs. Incidence and enteral feed antecedents of severe neonatal necrotising enterocolitis across neonatal networks in England, 2012-13: a whole-population surveillance study. Lancet Gastroenterol Hepatol. 2017;2(1):43-51.
41. Gale C, Jeyakumaran D, Longford N, Battersby C, Ojha S, Oughham K, et al. Administration of parenteral nutrition during therapeutic hypothermia: a population level observational study using routinely collected data held in the National Neonatal Research Database. Archives of Disease in Childhood - Fetal and Neonatal Edition. 2021;106(6):608.
42. Uthaya S, Longford N, Battersby C, Oughham K, Lanoue J, Modi N. Early versus later initiation of parenteral nutrition for very preterm infants: a propensity score-matched observational study. Archives of Disease in Childhood - Fetal and Neonatal Edition. 2021:fetalneonatal-2021-322383.
43. Webbe JWH, Longford N, Battersby C, Oughham K, Uthaya SN, Modi N, et al. Outcomes in relation to early parenteral nutrition use in preterm neonates born between 30 and 33 weeks' gestation: a propensity score matched observational study. Arch Dis Child Fetal Neonatal Ed. 2021.
44. Simmer K, Rao SC. Early introduction of lipids to parenterally-fed preterm infants. Cochrane Database Syst Rev. 2005(2):CD005256.

Confidential: For Review Only

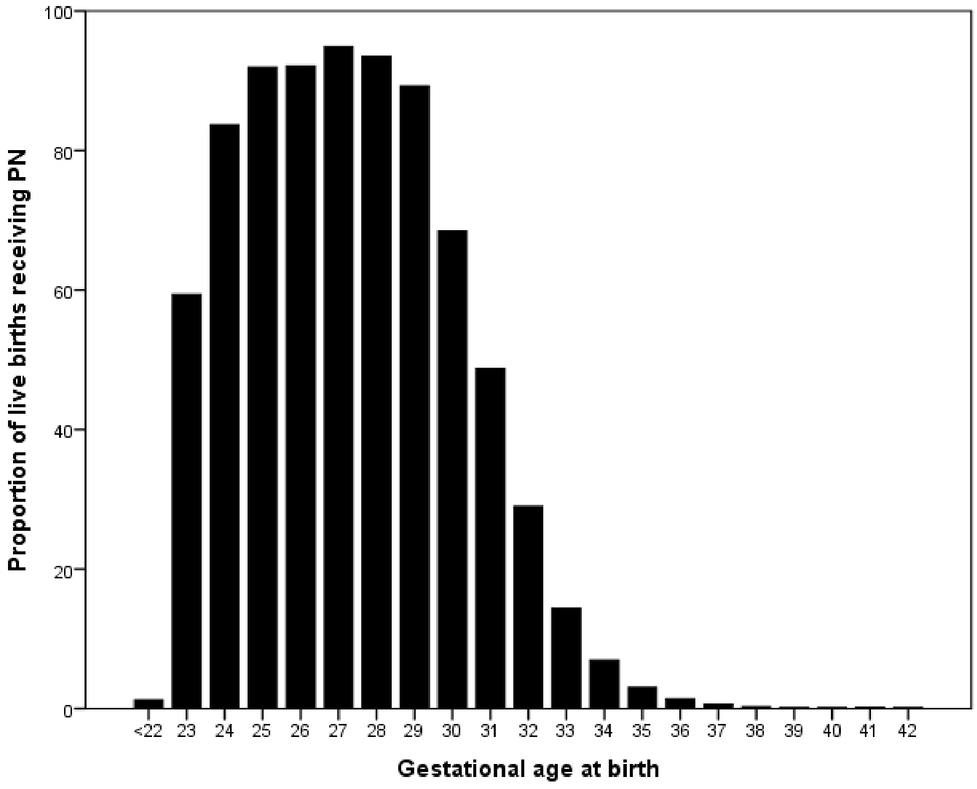

220x176mm (72 x 72 DPI)

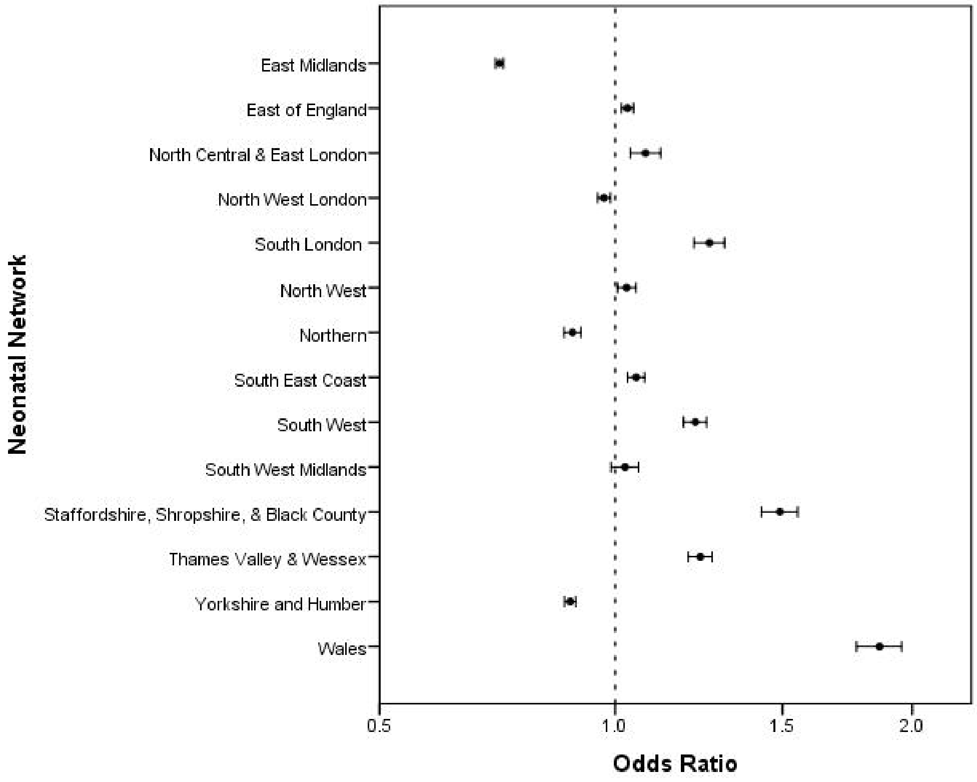

418x334mm (38 x 38 DPI)

Online only supplemental material

eTable 1: Data fields for extraction from NNRD
eTable 2: Gestational age of neonates receiving PN in the first postnatal week as proportion of neonatal unit admissions
eTable 3: Proportion of live births receiving PN in the first postnatal week by year of birth
eTable 4: Birth weight of neonates receiving PN in the first postnatal week as proportion of live births

Confidential: For Review Only

eTable 1: Data fields for extraction from NNRD

Outcome	
**Variable**	**Data items**
Parental nutrition	**PN group** defined as<br>Any of the following items entered in the 'Daily Care Fluids' and 'Feeding' during first 7 days:<br>• Y entry for PARENTERAL NUTRITION RECEIVED INDICATOR<br>Or<br>The following drug code entered in the Daily care medication during first 7 days:<br>• 1010238 Total parenteral nutrition<br><br>**No PN group** defined as<br>All other babies

Variables	
**Variable**	**Data items**
Gestational age at birth	Data extracted from GESTATION LENGTH (AT DELIVERY):
Birth weight	Data extracted from BIRTH WEIGHT
Neonatal network	Data extracted from SITE CODE (OF ACTUAL PLACE OF DELIVERY)

Confidential: For Review Only

eTable 2: Gestational age of neonates receiving PN in the first postnatal week as proportion of neonatal unit admissions

Gestational age category† at birth	Neonates receiving PN in the first postnatal week by year of birth					
	2012	2013	2014	2015	2016	2017
Extremely preterm	2317 *(92)*	2309 *(94)*	2267 *(94)*	2348 *(94)*	2421 *(96)*	2325 *(95)*
Very preterm	3493 *(65)*	3900 *(72)*	3896 *(73)*	4059 *(74)*	4135 *(75)*	4110 *(75)*
Moderate and late preterm	2343 *(9.3)*	2640 *(10)*	2796 *(11)*	2683 *(9.7)*	2547 *(9.2)*	2375 *(8.4)*
Term	1370 *(2.9)*	1688 *(3.4)*	1618 *(3.0)*	1528 *(2.7)*	1484 *(2.5)*	1493 *(2.3)*

†Gestational age at birth categorised using WHO definitions (33)
Extremely preterm: <28^{+0} weeks, Very preterm: 28^{+0} - 31^{+6} weeks, Moderate and late preterm; 32^{+0} - 36^{+6} weeks, Term >36^{+6} weeks
Number in brackets indicates the percentage of all live births given PN in each category (Denominator data from NNRD)

Confidential: For Review Only

eTable 3: Proportion of live births receiving PN in the first postnatal week by year of birth

Gestational age in weeks at birth	Neonates receiving PN in the first postnatal week by year of birth (%)						Trend analysis p value
	2012	2013	2014	2015	2016	2017	
<22	0.4	1.7	1.3	0.7	1.8	2.3	0.12
23	51	61	58	63	66	71.2	0.01
24	79	80	86	91	89	86	0.12
25	88	93	97	91	97	94	0.28
26	88	91	96	94	92	95	0.16
27	91	94	93	98	99	97	0.03
28	86	94	94	95	98	96	0.06
29	81	89	91	91	93	93	0.04
30	56	69	71	71	73	74	0.04
31	41	50	50	53	51	52	0.08
32	24	30	31	31	31	31	0.11
33	14	16	17	16	15	13	0.63
34	6.8	8.1	8.4	7.2	7.6	6.3	0.50
35	3.0	3.3	3.8	3.3	3.2	3.1	0.91
36	1.6	1.7	1.7	1.6	1.4	1.3	0.09
37	0.7	0.8	0.7	0.7	0.7	0.7	0.28
38	0.3	0.4	0.4	0.3	0.3	0.3	0.82
39	0.1	0.2	0.2	0.2	0.2	0.2	0.76
40	0.1	0.2	0.2	0.2	0.2	0.2	0.75
41	0.2	0.2	0.2	0.2	0.2	0.2	0.32
42	0.1	0.2	0.2	0.2	0.2	0.2	0.42

Denominator data from total number of live births from ONS Birth characteristics in England and Wales

Trend analysis for proportion of live births given PN in each category was completed: none of the trends over time were significant after Bonferroni-Holm correction for multiple comparisons

Neonates with missing data for gestational age = 3

Confidential: For Review Only

eTable 4: Birth weight of neonates receiving PN in the first postnatal week as proportion of live births

Birth weight category* at birth	Neonates receiving PN in the first postnatal week by year of birth						Trend analysis p value
	2012	2013	2014	2015	2016	2017	
<1kg	2227 *(67)*	2578 *(72)*	2594 *(73)*	2792 *(80)*	2724 *(82)*	2533 *(100)*†	0.01
<1.5kg	5375 *(70)*	5935 *(78)*	6054 *(81)*	6341 *(84)*	6393 *(88)*	6087 *(96)*	0.00
<2.5kg	7727 *(16)*	8359 *(18)*	8444 *(18)*	8883 *(18)*	8939 *(18)*	8629 *(18)*	0.06
>2.5kg	1290 (0.2)	1550 (0.3)	1504 (0.3)	1739 (0.3)	1655 (0.3)	1674 (0.3)	0.60

**Birth weight categorised using WHO definitions (34)*
Number in brackets indicates the percentage of all live births given PN in each category (Denominator data from ONS Birth characteristics in England and Wales)
Trend analysis for proportion of neonates admitted to a neonatal unit given PN in each category: p value was significant after Bonferroni-Holm correction for multiple comparisons for <1kg and <1.5kg categories
Neonates with missing data for birth weight = 4
†The ONS changed the way they clean birthweight data in 2017 and as a result the number of neonates born <1kg dropped from 3117 births in 2016 to 2538 in 2017 This change in the denominator inflated the proportion of <1kg births given PN in 2017.

Confidential: For Review Only

BMJ Paediatrics Open

Use of parenteral nutrition in the first postnatal week in England and Wales: An observational study using real-world data

Journal:	*BMJ Paediatrics Open*
Manuscript ID	bmjpo-2022-001543.R2
Article Type:	Original research
Date Submitted by the Author:	13-Jul-2022
Complete List of Authors:	Webbe, James; Imperial College London, Academic Neonatal Medicine<br>Battersby, Cheryl; Imperial College London, Medicine<br>Longford, Nicholas ; Imperial College London, Department of Medicine<br>Oughham , Kayleigh; Imperial College London Faculty of Medicine, Neonatal Data Analysis Unit<br>uthaya, s; Imperial College London, Neonatal Unit<br>Modi, Neena; Imperial College London, Neonatal Medicine<br>Gale, Chris; Imperial College London, Academic Neonatal Medicine
Keywords:	Neonatology, Epidemiology

SCHOLARONE™
Manuscripts

I, the Submitting Author has the right to grant and does grant on behalf of all authors of the Work (as defined in the below author licence), an exclusive licence and/or a non-exclusive licence for contributions from authors who are: i) UK Crown employees; ii) where BMJ has agreed a CC-BY licence shall apply, and/or iii) in accordance with the terms applicable for US Federal Government officers or employees acting as part of their official duties; on a worldwide, perpetual, irrevocable, royalty-free basis to BMJ Publishing Group Ltd ("BMJ") its licensees and where the relevant Journal is co-owned by BMJ to the co-owners of the Journal, to publish the Work in this journal and any other BMJ products and to exploit all rights, as set out in our licence.

The Submitting Author accepts and understands that any supply made under these terms is made by BMJ to the Submitting Author unless you are acting as an employee on behalf of your employer or a postgraduate student of an affiliated institution which is paying any applicable article publishing charge ("APC") for Open Access articles. Where the Submitting Author wishes to make the Work available on an Open Access basis (and intends to pay the relevant APC), the terms of reuse of such Open Access shall be governed by a Creative Commons licence – details of these licences and which Creative Commons licence will apply to this Work are set out in our licence referred to above.

Other than as permitted in any relevant BMJ Author's Self Archiving Policies, I confirm this Work has not been accepted for publication elsewhere, is not being considered for publication elsewhere and does not duplicate material already published. I confirm all authors consent to publication of this Work and authorise the granting of this licence.

Confidential: For Review Only

Use of parenteral nutrition in the first postnatal week in England and Wales: An observational study using real-world data

J Webbe[1], C Battersby[2], N Longford[3], K Ougham[4], S Uthaya[5], N Modi[6], C Gale[7]

[1] James Webbe, PhD

Neonatal Medicine, School of Public Health, Faculty of Medicine, Imperial College London

j.webbe@imperial.ac.uk

[2] Cheryl Battersby, PhD

Neonatal Medicine, School of Public Health, Faculty of Medicine, Imperial College London

c.battersby@imperial.ac.uk

[3] Nicholas Longford, PhD

Neonatal Medicine, School of Public Health, Faculty of Medicine, Imperial College London

n.longford@imperial.ac.uk

[4] Kayleigh Ougham, MSc

Neonatal Medicine, School of Public Health, Faculty of Medicine, Imperial College London

k.ougham@imperial.ac.uk

[5] Sabita Uthaya, MD

Neonatal Medicine, School of Public Health, Faculty of Medicine, Imperial College London

s.uthaya@imperial.ac.uk

[6] Neena Modi, FMedSci

Confidential: For Review Only

Neonatal Medicine, School of Public Health, Faculty of Medicine, Imperial College London

n.modi@imperial.ac.uk

[7] Chris Gale, PhD

Neonatal Medicine, School of Public Health, Faculty of Medicine, Imperial College London

christopher.gale@imperial.ac.uk

Corresponding author

Dr Christopher Gale, Reader in Neonatal Medicine

Neonatal Medicine, Chelsea and Westminster campus, Imperial College London

369 Fulham Road, London, SW10 9NX, United Kingdom.

+ 44 (0)203 3155418

christopher.gale@imperial.ac.uk

Manuscript word count: 2252

Confidential: For Review Only

ABSTRACT

Background

Parenteral nutrition (PN) is used to provide supplemental support to neonates while enteral feeding is being established. PN is a high-cost intervention with beneficial and harmful effects. Internationally there is substantial variation in how PN is used, and there are limited contemporary data describing use across the UK.

Objective

To describe PN use in the first postnatal week in infants born and admitted to neonatal care in England, Scotland and Wales.

Method

Data describing neonates admitted to National Health Service (NHS) neonatal units between 1st January 2012 and 31st December 2017, extracted from routinely recorded data held the National Neonatal Research Database (NNRD); the denominator was live births, from Office for National Statistics.

Results

Over the study period 62,145 neonates were given PN in the first postnatal week (1.4% of all live births); use was higher in more preterm neonates (76% of livebirths at <28 weeks, 0.2% of term livebirths) and in neonates with lower birth weight. 15% (9181/62145) of neonates given PN in the first postnatal week were born at term. There was geographic variation in PN administration: the proportion of live births given PN within neonatal regional networks ranged from 1.0% (95% confidence intervals: 1.0, 1.0) to 2.8% (95% confidence interval: 2.7, 2.9).

Conclusions and relevance

Significant variation exists in neonatal PN use; it is unlikely this reflects optimal use of an expensive intervention. Research is needed to identify which babies will benefit most and which are at risk of harm from early PN.

Registration

ClinicalTrials.gov: NCT03767634

Confidential: For Review Only

Registration date: 06/12/2018

Abstract word count: 250/250

Confidential: For Review Only

INTRODUCTION

In 1968 parenteral nutrition (PN) was used to support the metabolic needs of a term neonate with small bowel atresia (1). Following this, PN has been increasingly used to supplement the nutrition of sick or preterm neonates. The widespread use of PN has been encouraged on the basis that optimising nutrition will improve short and long-term outcomes (2). It is considered most beneficial for neonates born preterm or with lower birth weight who have fewer reserves and may accrue large nutritional deficits before enteral feeds are established (2). Despite widespread use, the impact of PN on key neonatal outcomes has not been evaluated in randomised controlled neonatal trials powered for clinically meaningful and functional end-points.

Therefore, while effects on short-term biochemical markers such as nitrogen balance are well described (3), evidence to support beneficial effects on survival and neurodevelopment are lacking (3, 4). Conversely, PN carries well described risks, of which the most serious and common is bloodstream infection (5). Recent evidence from large randomised controlled trials in critically unwell adults (6) and children (7) showed that use of PN during the first seven days of admission to an intensive care unit led to worse outcomes, when compared to delayed PN administration, indicating that the harms of early PN outweigh benefits in these populations. Although there has not been a similar trial in neonatal care, subgroup analysis in the PePaNIC trial of term neonates looked after on paediatric intensive care units also showed increased rates of nosocomial infection with early PN use (8), suggesting that early PN use should be targeted at neonates with most potential for benefit.

Given the uncertain balance of risk and benefit for neonatal PN use, it is unsurprising that international practice is variable: some neonatal units in high-income countries provide PN to up to 70% of neonatal admissions (9) while others report not using PN (10). In the United Kingdom, a 2011 report from the National Confidential Enquiry into Patient Outcome and Death found considerable variation in neonatal management in 2008 with only 24% of patients receiving PN that was considered best practice (11). Following this, a national

Confidential: For Review Only

framework (12), and NICE guidance (13) have been developed. The most recent NICE guidance makes recommendations about prescription, administration, monitoring and recipients of PN in neonatal units; recommending all neonates born before 30^{+0} $weeks^{+days}$ gestation or weighing under 1250g at birth, and any who are unable (or not expected) to establish milk feeds of ≥100mL/kg/day by postnatal day five, receive early PN.

We aimed to describe how PN is used in the first postnatal week, to explore how use is influenced by gestational age, birth weight, geographical region, and to compare how use has changed over time in the period prior to the publication of 2020 NICE guidance.

OBJECTIVE

To describe the pattern of PN use in neonatal units in England, Scotland and Wales in the first seven postnatal days.

METHODS

Study design

This study was an epidemiological description of practice: we pre-registered it (Clinicaltrials.gov: NCT03767634) and published the study protocol (14). We report it in line with RECORD guidelines (15).

Data source

We used de-identified data held in the National Neonatal Research Database (NNRD) (16). The NNRD holds data extracted from electronic health records completed by health professionals during routine clinical care (17). The Neonatal Data Set, a defined national data standard (18) comprising approximately 450 items, is extracted and transmitted to the Neonatal Data Analysis Unit at Imperial College London. The NNRD holds data from all neonates admitted to NHS neonatal units in England, Scotland and Wales; in total the NNRD contains data from about one million neonates from 2008 to the present. Accuracy and

Confidential: For Review Only

completeness of NNRD data have been confirmed by comparison with Case Record Forms from a prospective clinical trial, which showed high data completeness and accuracy (>95%) (19). The Chief Investigator of the NNRD (NM) holds UK Research Ethics Committee approval (REC Reference: 16/LO/1093), and Confidentiality Advisory Group approval (ECC 8-05(f/2010)) for the NNRD as a research database. Study specific Research Ethics Committee (18/NI/0214), Health Research Authority and Health and Care Research Wales approval and approval for inclusion of their data in this study from all English neonatal units was obtained. Data for this study was extracted by author KO, operating within the guidelines established by these approvals; no other investigators had accessed the wider NNRD for this study. No data cleaning methods were required for this study, and no data linkage was required. We obtained population-level data for total live births by gestational age and birth weight from the Office for National Statistics (ONS) (20-25), and for live births by neonatal network from Mothers and Babies: Reducing Risk through Audits and Confidential Enquiries across the UK (MBRRACE-UK) reports (26-29), for denominator data. Population-level data for total neonatal unit admissions by gestational age and birth weight were obtained from the NNRD for denominator data.

Participants

The study population was all neonates born between 1st January 2012 and 31st December 2017 and admitted to a neonatal unit in England and Wales. There were no exclusion criteria: this was intended to maximise the sample size and ensure complete, population-level data.

Variables

The primary outcome was any use of PN in the first seven postnatal days. To describe the background characteristics of neonates given PN we extracted data relating to gestational age at birth, birth weight, year of birth, and neonatal network (30) of birth. The variables extracted from the NNRD, and how they were defined, are listed in eTable 1.

Statistical methods

https://mc.manuscriptcentral.com/bmjpo

Confidential: For Review Only

We described the characteristics of neonates that received PN in the first seven postnatal days and compared use between different groups. For gestational age and birth weight, we grouped neonates according to well-established and widely accepted WHO categories (31)(32). To explore differences in PN use across geographical regions, we grouped neonates by neonatal network of birth (30). We compared changes in PN prescribing over time by grouping neonates according to the year of birth.

RESULTS

The only deviation from the protocol related to the use of data from neonatal units in Scotland. We were unable to use data on babies from Scottish neonatal units due to difficulties obtaining the institutional approvals required for Public Benefit and Privacy Panel for Health and Social Care (33) approval. The project was completed using data from neonatal units in England and Wales only.

Over the six-year study period 4,196,314 neonates were born in England and Wales. Of these, 347,959 neonates were admitted to NHS neonatal units and had data held within the NNRD; 62,145 were recorded as having received PN during the first postnatal week. This equates to just over 1% of all live births (Table 1) and 17% of all neonatal unit admissions (eTable 2). PN was given to neonates of all gestational ages, with neonates born preterm more likely to be recipients. However, a large proportion of the neonates who receive PN were more mature: 15% of neonates who received PN in the first postnatal week over the study period were born at term.

Table 1 Gestational age of neonates receiving PN in the first postnatal week as a proportion of total live births

Gestational age category* at birth	Neonates receiving PN in the first postnatal week by year of birth n (% of live births)					
	2012	2013	2014	2015	2016	2017
Extremely preterm	2317 *(72)*	2309 *(75)*	2267 *(77)*	2348 *(78)*	2421 *(76)*	2325 *(75)*

Confidential: For Review Only

Very preterm	3493 *(61)*	3900 *(71)*	3896 *(71)*	4059 *(73)*	4135 *(73)*	4110 *(74)*
Moderate and late preterm	2343 *(5.3)*	2640 *(6.1)*	2796 *(6.4)*	2683 *(6.0)*	2547 *(5.6)*	2375 *(5.2)*
Term	1370 *(0.2)*	1688 *(0.3)*	1618 *(0.3)*	1528 *(0.2)*	1484 *(0.2)*	1493 *(0.2)*
Total	9523 *(1.3)*	10537 *(1.5)*	10577 *(1.5)*	10618 *(1.5)*	10587 *(1.5)*	10303 *(1.5)*

**Gestational age at birth categorised using WHO definitions (31)*
Extremely preterm: <28^{+0} weeks, Very preterm: 28^{+0} - 31^{+6} weeks, Moderate and late preterm; 32^{+0} - 36^{+6} weeks, Term >36^{+6} weeks
Number in brackets indicates the percentage of all live births given PN in each category (Denominator data from ONS Birth characteristics in England and Wales)
Neonates with missing data for gestational age = 3

The proportion of live births who receive PN in the first postnatal week is lower in neonates born at 22 or 23 weeks, rises to over 90% for neonates born from 25 to 28 weeks gestation before falling again (Figure 1), (eTable 3).

Neonates born with lower birth weights were more likely to receive PN in the first postnatal week (Table 2, eTable 4).

Table 2 Birth weight of neonates receiving PN in the first postnatal week as proportion of neonatal unit admissions

Birth weight at birth*	Neonates receiving PN in the first postnatal week by year of birth n (% of neonatal units admissions)					
	2012	2013	2014	2015	2016	2017
<1kg	2401 *(92)*	2484 *(94)*	2442 *(95)*	2542 *(95)*	2547 *(96)*	2541 *(96)*
<1.5kg	5591 *(77)*	5878 *(82)*	5936 *(83)*	6090 *(84)*	6213 *(85)*	6095 *(85)*
<2.5kg	7999 *(27)*	8679 *(29)*	8756 *(29)*	8880 *(28)*	8935 *(28)*	8628 *(28)*
>2.5kg	1524 *(3.1)*	1857 *(3.5)*	1821 *(3.2)*	1738 *(2.8)*	1652 *(2.6)*	1675 *(2.4)*

**Birth weight categorised using WHO definitions (32)*
Number in brackets indicates the percentage of all neonates admitted to a neonatal unit given PN in each category (Denominator data from NNRD)
Neonates with missing data for birth weight = 4

Confidential: For Review Only

The use of PN differed across networks, with a range from 1.0% to 2.8% of all live births given PN in the first postnatal week (Figure 2)(Table 3). Rates of PN administration within neonatal networks varied less over time than the differences seen between networks.

Table 3 Neonates receiving PN in the first postnatal week by Neonatal Network as proportion of live births

Neonatal Network at birth	Babies receiving PN in the first postnatal week by year of birth n (% of live births)					
	2012	2013	2014	2015	2016	2017
East Midlands[1]	608 *(†)*	601 *(1.1%)*	607 *(1.1%)*	553 *(1.0%)*	570 *(1.0%)*	566 *(1.0%)*
East of England	953 *(†)*	973 *(1.4%)*	1052 *(1.5%)*	942 *(1.4%)*	961 *(1.4%)*	926 *(1.4%)*
London - North Central & East	771 *(†)*	733 *(1.4%)*	806 *(1.5%)*	842 *(1.6%)*	845 *(1.6%)*	845 *(1.6%)*
London - North West	417 *(†)*	429 *(1.4%)*	421 *(1.3%)*	403 *(1.3%)*	443 *(1.4%)*	409 *(1.3%)*
London - South	725 *(†)*	723 *(1.6%)*	739 *(1.7%)*	804 *(1.8%)*	828 *(1.8%)*	764 *(1.7%)*
North West	1233 *(†)*	1206 *(1.5%)*	1222 *(1.5%)*	1231 *(1.5%)*	1130 *(1.3%)*	1031 *(1.2%)*
Northern	326 *(†)*	398 *(1.2%)*	397 *(1.2%)*	408 *(1.3%)*	413 *(1.3%)*	363 *(1.1%)*
South East Coast	681 *(†)*	723 *(1.5%)*	707 *(1.5%)*	720 *(1.5%)*	717 *(1.5%)*	710 *(1.5%)*
South West	849 *(†)*	805 *(1.7%)*	785 *(1.6%)*	839 *(1.8%)*	813 *(1.7%)*	786 *(1.6%)*
Southern West Midlands	425 *(†)*	426 *(1.3%)*	443 *(1.4%)*	431 *(1.4%)*	484 *(1.5%)*	454 *(1.5%)*
Staffordshire, Shropshire, & Black Country	495 *(†)*	534 *(2.1%)*	527 *(2.1%)*	553 *(2.3%)*	479 *(1.9%)*	499 *(2.0%)*
Thames Valley & Wessex	1062 *(†)*	1068 *(1.9%)*	1021 *(1.7%)*	1052 *(1.8%)*	1045 *(1.7%)*	1034 *(1.8%)*
Wales	166 *(†)*	933 *(2.9%)*	910 *(2.9%)*	881 *(2.8%)*	846 *(2.7%)*	888 *(2.8%)*
Yorkshire & Humber	692 *(†)*	820 *(1.2%)*	791 *(1.2%)*	795 *(1.2%)*	847 *(1.3%)*	878 *(1.3%)*

[1]East Midlands Operational Delivery Network previously the separate Central and Trent Networks
Number in brackets indicates the percentage of all live births given PN in each Neonatal Network (Data from MBRRACE-UK reports)
†MBRRACE-UK report not produced for 2012
Neonates with missing data for neonatal network = 202

DISCUSSION

Confidential: For Review Only

In this work, we described the characteristics of neonates born in England and Wales who receive PN in the first postnatal week. PN is a common intervention on neonatal units and is given to 17% of all admissions. While PN use is higher in neonates born prematurely and with lower birth weight, a considerable proportion are born at term. We also show that use varies between different neonatal networks.

That higher rates of PN use is seen in neonates born more preterm is unsurprising, as these populations are considered most likely to benefit. The lower rates seen in the most preterm neonates (those born at <26 gestational weeks) are likely because many of these babies die before admission to a neonatal unit and before PN is commenced. However, because of the much larger proportion of babies born at more mature gestations it is noteworthy that around 15% of all babies given PN are born at term (although this still means that only 0.2% of term births receive PN). The energy requirements (34), indications for use and metabolic stability of these groups differs, thus the risks and benefits of PN may also differ substantially across gestational ages: guidelines and practice appropriate in one group may not be optimal in differing populations.

We found PN use in the first postnatal week varied significantly between neonatal networks. This is in keeping with variations between neonatal units in PN use in other settings (9, 10). However, we were unable to find another comprehensive population based study of national PN use. From our descriptive analysis, it is not possible to determine whether the variation we identified is explained by regional differences in rates of prematurity, neonatal sickness or other case-mix factors (such as congenital anomalies or neonatal surgical conditions), nor, given the paucity of evidence, is it possible to comment on what rate of PN use should be expected. However, by identifying wide variation in practice within the United Kingdom, we highlight early PN use as an area where optimal practice is uncertain (35). This variation also has financial implications. Treating a neonate with PN for one day in 2012 cost £37.43 (36), and if all hospitals in England and Wales treated the same proportion of live births as the

https://mc.manuscriptcentral.com/bmjpo

Confidential: For Review Only

highest use network this would cost the NHS an additional £2.5 million each year. This expense may be justified if PN leads to lower mortality or morbidity, but such evidence is lacking.

The strengths of our study include the population-level coverage involving a cohort of over 4 million neonates. The population-level data meant that recruitment bias was reduced, but not fully eliminated. The NNRD covers all neonatal units in England and Wales but does not include Paediatric Intensive Care Units and surgical units that admit neonates. Some term cardiac and surgical babies will be cared for on these units and thus we may have underestimated the amount of PN used in these groups. We followed a pre-specified protocol and data analysis plan and limited the risk of false discovery associated with multiple comparisons (37) by using the Holm-Bonferroni method. In keeping with previous studies that have used the NNRD, we had very little missing data (19, 38). Limitations of this study include that we were not able to obtain permission to use data for neonates born in Scotland, reducing the study population. As the NNRD only holds data for neonates admitted to an NHS neonatal unit for denominator data, we used data from the ONS and MBRRACE-UK to provide total numbers of live births. As MBRRACE-UK did not produce a report in 2012 this limited the number of years for which we could undertake network level comparisons. As has been found in previous studies, due to the lack of additional information about PN in the data extracted we are unable to describe changes in how PN was used (e.g. when PN was commenced or types of PN used) (39-41).

Our findings show that PN use in the first postnatal week is common in England and Wales, with regional variation. In light of the potential harm found with early PN in critically unwell children (7), and the lack of evidence of benefit for clinically important endpoints in infants (3, 42) research is needed to ensure that PN use in neonates is underpinned by a robust evidence base. Our data will help in the planning of future trials to identify which neonates will benefit from early PN and ensure that clinical practice is based on strong evidence. Approximately twenty-eight neonates are started on PN each day in England and Wales: if all of these diverse

Confidential: For Review Only

patients are to receive optimal treatment urgent research is needed to ensure they are the neonates who will benefit most from this intervention, and to avoid harm in others.

CONCLUSION

Parenteral nutrition is commonly used in the first postnatal week across the United Kingdom, with higher use in neonates born more preterm. Across all gestational age categories no change in PN use in the first postnatal week over time was found, but there is persisting variation in use between regional neonatal networks. Research is needed to ensure PN use in this group is well targeted.

Confidential: For Review Only

ABBREVIATIONS

ELBW	Extremely Low Birth Weight
LBW	Low Birth Weight
MBRRACE-UK	Mothers and Babies: Reducing Risk through Audits and Confidential Enquiries across the UK
NNRD	National Neonatal Research Database
ONS	Office for National Statistics
PICU	Paediatric Intensive Care Unit
PN	Parenteral Nutrition
VLBW	Very Low Birth Weight

Confidential: For Review Only

DECLARATIONS

Consent for publication

Not applicable

Availability of data and material

Applications to use the data used within this project should be made to the Neonatal Data Analysis Unit, Imperial College London

Competing interests

JW has received support from Chiesi Pharmaceuticals to attend an educational conference and has received a research grant from Mason Medical Research Foundation.

SU has received funding from the National Institute of Health Research, the Department of Health and Prolacta Life Sciences. SU has been on the Advisory Board of Fresenius Kabi and received honoraria and travel expenses for speaking at study days organised by Fresenius Kabi. SU is a member of the National Institute for Health and Care Excellence Parenteral Nutrition Guideline Development Committee. NM is the Chief Investgator for the National Neonatal Research Database. In the last five years NM has served on the Board of Trustees of the Royal College of Paediatrics and Child Health, David Harvey Trust, Medical Women's Federation, Medact, Action Cerebral Palsy, TheirWorld and Academy of Medical Sciences. NM is a member of the Nestle Scientific Advisory Board but accepts no personal remuneration for this role. NM has received research grants from the British Heart Foundation, Medical Research Council, National Institute of Health Research, Westminster Research Fund, Prolacta Life Sciences, Chiesi, Takeda, March of Dimes, Health Data Research UK, HCA International, and European Health Data Evidence Network; travel and accommodation expenses from Prolacta, Nestle and Chiesi.

Confidential: For Review Only

CG has received support from Chiesi Pharmaceuticals to attend an educational conference; in the past 5 years he been investigator on received research grants from Medical Research Council, National Institute of Health Research, Canadian Institute of Health Research, Department of Health in England, Mason Medical Research Foundation, Westminster Medical School Research Trust and Chiesi Pharmaceuticals.

CB has received support from Chiesi Pharmaceuticals to attend an educational conference; she is supported by a personal award by the National Institute of Health Research.

Funding

The NNRD is created and maintained through grants and unrestricted funding held by NM. This includes costs involved in data transfer, storage, cleaning, merging, administration and regulatory approvals. The extraction of study data from the NNRD and analysis for this study was funded through a Mason Medical Research Fellowship awarded to JW. CG is funded by the United Kingdom Medical Research Council (MRC) through a Clinician Scientist Fellowship award. The MRC and Mason Medical Research Foundation were not involved in the design of the study, collection, analysis, and interpretation of data or in writing the manuscript

Authors' contributions

JW, CG and NM conceived this project. JW, CG, KO and NL planned the statistical analyses. The first draft of the manuscript was written by JW and revised by NM; CG, SU, CB, NL and NM edited and reviewed the manuscript. It was approved by JW, CG, CB, KO, NL, SU and NM.

Acknowledgements

We wish to thank Angela Richard-Löndt and Laura Noakes, parents of preterm infants, for their support in developing this research project and BLISS for their input and support developing the dissemination plan.

Confidential: For Review Only

What is already known on this topic?

- Parenteral nutrition is commonly given to neonates unable to receive adequate milk feeds or while milk feeds are introduced
- There is limited evidence to inform parenteral nutrition use in neonates

What this study adds?

- The early use of parenteral nutrition is common; 17% of all babies admitted to neonatal units receive PN in the first postnatal week
- Fifteen percent of neonates given PN in the first postnatal week are born full-term
- There is variation in the use of parenteral nutrition in the first week of postnatal life between regional neonatal networks

Confidential: For Review Only

REFERENCES

1. Wilmore DW, Dudrick SJ. Growth and Development of an Infant Receiving All Nutrients Exclusively by Vein. Jama. 1968;203(10):860-4.
2. Ziegler EE, Thureen PJ, Carlson SJ. Aggressive nutrition of the very low birthweight infant. Clinics in perinatology. 2002;29(2):225-44.
3. Trivedi A, Sinn JK. Early versus late administration of amino acids in preterm infants receiving parenteral nutrition. Cochrane Database Syst Rev. 2013(7):CD008771.
4. Moyses HE, Johnson MJ, Leaf AA, Cornelius VR. Early parenteral nutrition and growth outcomes in preterm infants: a systematic review and meta-analysis. The American journal of clinical nutrition. 2013;97(4):816-26.
5. Zingg W, Tomaske M, Martin M. Risk of parenteral nutrition in neonates--an overview. Nutrients. 2012;4(10):1490-503.
6. Casaer MP, Mesotten D, Hermans G, Wouters PJ, Schetz M, Meyfroidt G, et al. Early versus late parenteral nutrition in critically ill adults. N Engl J Med. 2011;365(6):506-17.
7. Fivez T, Kerklaan D, Mesotten D, Verbruggen S, Wouters PJ, Vanhorebeek I, et al. Early versus Late Parenteral Nutrition in Critically Ill Children. N Engl J Med. 2016;374(12):1111-22.
8. van Puffelen E, Vanhorebeek I, Joosten KFM, Wouters PJ, Van den Berghe G, Verbruggen S. Early versus late parenteral nutrition in critically ill, term neonates: a preplanned secondary subgroup analysis of the PEPaNIC multicentre, randomised controlled trial. The Lancet Child & adolescent health. 2018;2(7):505-15.
9. Christensen RD, Henry E, Wiedmeier SE, Burnett J, Lambert DK. Identifying patients, on the first day of life, at high-risk of developing parenteral nutrition-associated liver disease. Journal of perinatology : official journal of the California Perinatal Association. 2007;27(5):284-90.
10. Ksiazyk J, Swietlinski J, Brozek G, Musialik-Swietlinska E, Migdal M, Zejda JE. [Parenteral nutrition in neonatal units: a survey of current practices in Poland]. Medycyna wieku rozwojowego. 2008;12(4 Pt 1):899-904.
11. Mason DG, Puntis JWL, McCormick K, Smith N. Parenteral nutrition for neonates and children: a mixed bag. Archives of Disease in Childhood. 2011;96(3):209.
12. British Association of Perinatal Medicine. The Provision of Parenteral Nutrition within Neonatal Services - A Framework for Practice [Guideline]. 2016 [updated April 2016. Available from: http://www.bapm.org/publications/documents/guidelines/Parenteral%20Nutrition%20April%202016.pdf.
13. Excellence; NIfHaC. Neonatal parenteral nutrition: NICE guideline [NG154]. 2020.
14. Webbe J, Longford N, Uthaya S, Modi N, Gale C. Outcomes following early parenteral nutrition use in preterm neonates: protocol for an observational study. BMJ open. 2019;9(7):e029065.
15. Benchimol EI, Smeeth L, Guttmann A, Harron K, Moher D, Petersen I, et al. The REporting of studies Conducted using Observational Routinely-collected health Data (RECORD) Statement. PLOS Medicine. 2015;12(10):e1001885.
16. Gale C, Morris I. The UK National Neonatal Research Database: using neonatal data for research, quality improvement and more. Archives of disease in childhood Education and practice edition. 2016;101(4):216-8.
17. Spencer A, Modi N. National neonatal data to support specialist care and improve infant outcomes. Arch Dis Child Fetal Neonatal Ed. 2013;98(2):F175-80.
18. NHS Digital. National Neonatal Data Set 2017 [Available from: http://www.datadictionary.nhs.uk/data_dictionary/messages/clinical_data_sets/data_sets/national_neonatal_data_set/national_neonatal_data_set_-_episodic_and_daily_care_fr.asp?shownav=1.

Confidential: For Review Only

19. Battersby C, Statnikov Y, Santhakumaran S, Gray D, Modi N, Costeloe K, et al. The United Kingdom National Neonatal Research Database: A validation study. PloS one. 2018;13(8).
20. Office for National Statistics. Characteristics of Birth 1, England and Wales: 2012. Fareham: Vital Statistics Outputs Branch, Office for National Statistics; 2013.
21. Office for National Statistics. Characteristics of Birth 1, England and Wales: 2013. Fareham: Vital Statistics Outputs Branch, Office for National Statistics; 2014.
22. Office for National Statistics. Birth Characteristics in England and Wales: 2014. Fareham: Vital Statistics Outputs Branch, Office for National Statistics; 2015.
23. Office for National Statistics. Birth Characteristics in England and Wales: 2015. Fareham: Vital Statistics Outputs Branch, Office for National Statistics; 2016.
24. Office for National Statistics. Birth Characteristics in England and Wales: 2016. Fareham: Vital Statistics Outputs Branch, Office for National Statistics; 2017.
25. Office for National Statistics. Birth Characteristics, England and Wales: 2017. Fareham: Vital Statistics Outputs Branch, Office for National Statistics; 2018.
26. Draper ES, Gallimore ID, Kurinczuk JJ, Smith PW, Boby T, Smith LK, et al. MBRRACE-UK Perinatal Mortality Surveillance Report, UK Perinatal Deaths for Births from January to December 2016. Leicester: The Infant Mortality and Morbidity Studies, Department of Health Sciences, University of Leicester; 2018.
27. Manktelow BN, Smith LK, Prunet C, Smith PW, Boby T, Hyman-Taylor P, et al. MBRRACE-UK Perinatal Surveillance Report: UK Perinatal Deaths for Births from January to December 2015. Leicester: The Infant Mortality and Morbidity Studies, Department of Health Sciences, University of Leicester; 2017.
28. Manktelow BN, Smith LK, Seaton SE, Hyman-Taylor P, Kurinczuk JJ, Field DJ, et al. MBRRACE-UK Perinatal Mortality Surveillance Report, UK Perinatal Deaths for Births from January to December 2014. Leicester: The Infant Mortality and Morbidity Studies, Department of Health Sciences, University of Leicester; 2016.
29. Manktelow BN, Smith LK, Evans TA, Hyman-Taylor P, Kurinczuk JJ, Field DJ, et al. Perinatal Mortality Surveillance Report UK Perinatal Deaths for births from January to December 2013. Leicester: The Infant Mortality and Morbidity Group, Department of Health Sciences, University of Leicester; 2015.
30. Gale C, Santhakumaran S, Nagarajan S, Statnikov Y, Modi N, Unit NDA, et al. Impact of managed clinical networks on NHS specialist neonatal services in England: population based study. Brit Med J. 2012;344.
31. Blencowe H, Cousens S, Chou D, Oestergaard M, Say L, Moller AB, et al. Born Too Soon: The global epidemiology of 15 million preterm births. Reprod Health. 2013;10.
32. World Health Organization. International Statistical Classification of Diseases and Related Health Problems 10th Revision. 2016.
33. Morris C, Gray A, Scott J, editors. NHS Scotland Public Benefit and Privacy Panel (PBPP) – Much Ado about Governance 3 years on. International Population Data Linkage Conference; 2018; Banff, Canada.
34. Schanler RJ, Garza C, Nichols BL. Fortified mothers' milk for very low birth weight infants: results of growth and nutrient balance studies. J Pediatr. 1985;107(3):437-45.
35. Soll RF, McGuire W. Evidence-Based Practice: Improving the Quality of Perinatal Care. Neonatology. 2019:1-6.
36. Walter E, Liu FX, Maton P, Storme T, Perrinet M, von Delft O, et al. Cost analysis of neonatal and pediatric parenteral nutrition in Europe: a multi-country study. European Journal Of Clinical Nutrition. 2012;66:639.
37. Young SS, Karr A. Deming, data and observational studies. Significance. 2011;8(3):116-20.
38. Battersby C, Longford N, Mandalia S, Costeloe K, Modi N, group UKNCNEs. Incidence and enteral feed antecedents of severe neonatal necrotising enterocolitis across neonatal networks in England, 2012-13: a whole-population surveillance study. Lancet Gastroenterol Hepatol. 2017;2(1):43-51.

Confidential: For Review Only

39. Gale C, Jeyakumaran D, Longford N, Battersby C, Ojha S, Oughham K, et al. Administration of parenteral nutrition during therapeutic hypothermia: a population level observational study using routinely collected data held in the National Neonatal Research Database. Archives of Disease in Childhood - Fetal and Neonatal Edition. 2021;106(6):608.
40. Uthaya S, Longford N, Battersby C, Oughham K, Lanoue J, Modi N. Early versus later initiation of parenteral nutrition for very preterm infants: a propensity score-matched observational study. Archives of Disease in Childhood - Fetal and Neonatal Edition. 2021:fetalneonatal-2021-322383.
41. Webbe JWH, Longford N, Battersby C, Oughham K, Uthaya SN, Modi N, et al. Outcomes in relation to early parenteral nutrition use in preterm neonates born between 30 and 33 weeks' gestation: a propensity score matched observational study. Arch Dis Child Fetal Neonatal Ed. 2021.
42. Simmer K, Rao SC. Early introduction of lipids to parenterally-fed preterm infants. Cochrane Database Syst Rev. 2005(2):CD005256.

Confidential: For Review Only

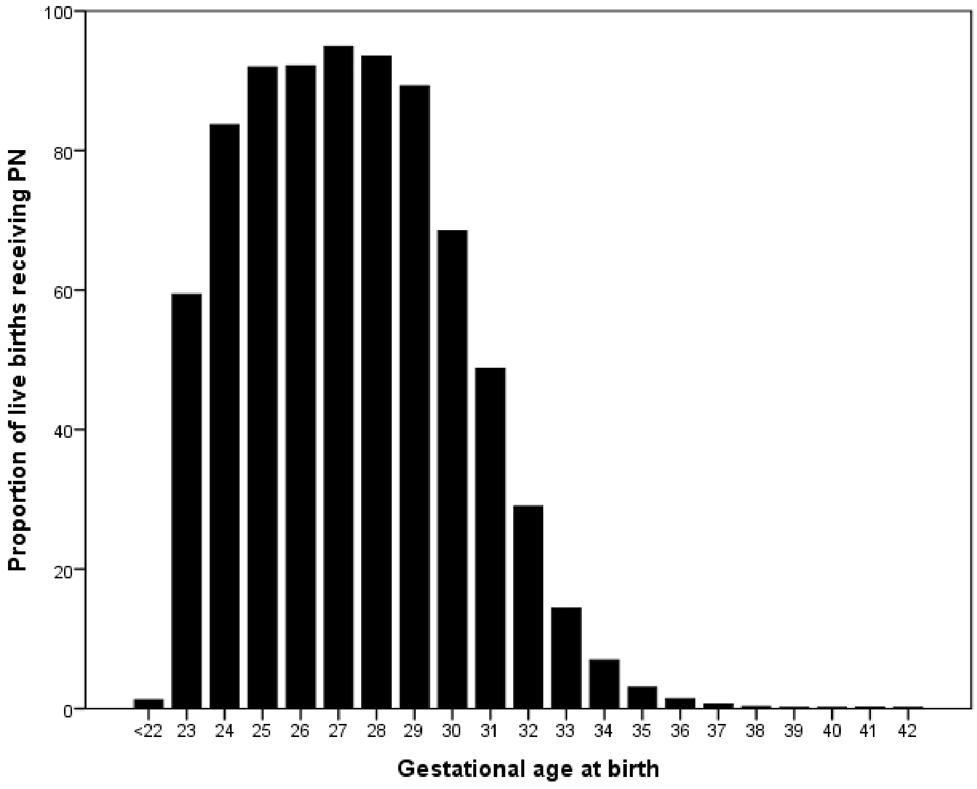

220x176mm (72 x 72 DPI)

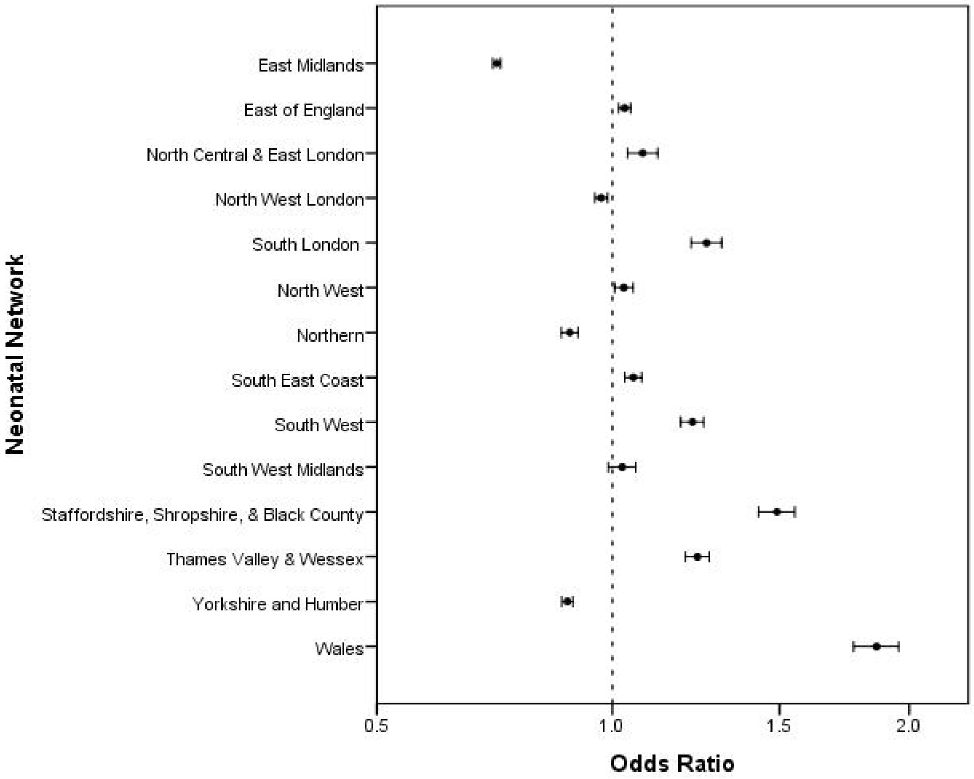

418x334mm (38 x 38 DPI)

Online only supplemental material

eTable 1: Data fields for extraction from NNRD
eTable 2: Gestational age of neonates receiving PN in the first postnatal week as proportion of neonatal unit admissions
eTable 3: Proportion of live births receiving PN in the first postnatal week by year of birth
eTable 4: Birth weight of neonates receiving PN in the first postnatal week as proportion of live births

Confidential: For Review Only

eTable 1: Data fields for extraction from NNRD

Outcome	
**Variable**	**Data items**
Parenteral nutrition	**PN group** defined as<br>Any of the following items entered in the 'Daily Care Fluids' and 'Feeding' during first 7 days:<br>• Y entry for PARENTERAL NUTRITION RECEIVED INDICATOR<br>Or<br>The following drug code entered in the Daily care medication during first 7 days:<br>• 1010238 Total parenteral nutrition<br><br>**No PN group** defined as<br>All other babies

Variables	
**Variable**	**Data items**
Gestational age at birth	Data extracted from GESTATION LENGTH (AT DELIVERY):
Birth weight	Data extracted from BIRTH WEIGHT
Neonatal network	Data extracted from SITE CODE (OF ACTUAL PLACE OF DELIVERY)

Confidential: For Review Only

eTable 2: Gestational age of neonates receiving PN in the first postnatal week as proportion of neonatal unit admissions

Gestational age category† at birth	Neonates receiving PN in the first postnatal week by year of birth					
	2012	2013	2014	2015	2016	2017
Extremely preterm	2317 *(92)*	2309 *(94)*	2267 *(94)*	2348 *(94)*	2421 *(96)*	2325 *(95)*
Very preterm	3493 *(65)*	3900 *(72)*	3896 *(73)*	4059 *(74)*	4135 *(75)*	4110 *(75)*
Moderate and late preterm	2343 *(9.3)*	2640 *(10)*	2796 *(11)*	2683 *(9.7)*	2547 *(9.2)*	2375 *(8.4)*
Term	1370 *(2.9)*	1688 *(3.4)*	1618 *(3.0)*	1528 *(2.7)*	1484 *(2.5)*	1493 *(2.3)*

†Gestational age at birth categorised using WHO definitions (33)
Extremely preterm: <28^{+0} weeks, Very preterm: 28^{+0} - 31^{+6} weeks, Moderate and late preterm; 32^{+0} - 36^{+6} weeks, Term >36^{+6} weeks
Number in brackets indicates the percentage of all live births given PN in each category (Denominator data from NNRD)

Confidential: For Review Only

eTable 3: Proportion of live births receiving PN in the first postnatal week by year of birth

Gestational age in weeks at birth	Neonates receiving PN in the first postnatal week by year of birth (%)						Trend analysis p value
	2012	2013	2014	2015	2016	2017	
<22	0.4	1.7	1.3	0.7	1.8	2.3	0.12
23	51	61	58	63	66	71.2	0.01
24	79	80	86	91	89	86	0.12
25	88	93	97	91	97	94	0.28
26	88	91	96	94	92	95	0.16
27	91	94	93	98	99	97	0.03
28	86	94	94	95	98	96	0.06
29	81	89	91	91	93	93	0.04
30	56	69	71	71	73	74	0.04
31	41	50	50	53	51	52	0.08
32	24	30	31	31	31	31	0.11
33	14	16	17	16	15	13	0.63
34	6.8	8.1	8.4	7.2	7.6	6.3	0.50
35	3.0	3.3	3.8	3.3	3.2	3.1	0.91
36	1.6	1.7	1.7	1.6	1.4	1.3	0.09
37	0.7	0.8	0.7	0.7	0.7	0.7	0.28
38	0.3	0.4	0.4	0.3	0.3	0.3	0.82
39	0.1	0.2	0.2	0.2	0.2	0.2	0.76
40	0.1	0.2	0.2	0.2	0.2	0.2	0.75
41	0.2	0.2	0.2	0.2	0.2	0.2	0.32
42	0.1	0.2	0.2	0.2	0.2	0.2	0.42

Denominator data from total number of live births from ONS Birth characteristics in England and Wales
Trend analysis for proportion of live births given PN in each category was completed: none of the trends over time were significant after Bonferroni-Holm correction for multiple comparisons
Neonates with missing data for gestational age = 3

Confidential: For Review Only

eTable 4: Birth weight of neonates receiving PN in the first postnatal week as proportion of live births

Birth weight category* at birth	Neonates receiving PN in the first postnatal week by year of birth						Trend analysis p value
	2012	2013	2014	2015	2016	2017	
<1kg	2227 *(67)*	2578 *(72)*	2594 *(73)*	2792 *(80)*	2724 *(82)*	2533 *(100)*†	0.01
<1.5kg	5375 *(70)*	5935 *(78)*	6054 *(81)*	6341 *(84)*	6393 *(88)*	6087 *(96)*	0.00
<2.5kg	7727 *(16)*	8359 *(18)*	8444 *(18)*	8883 *(18)*	8939 *(18)*	8629 *(18)*	0.06
>2.5kg	1290 (0.2)	1550 (0.3)	1504 (0.3)	1739 (0.3)	1655 (0.3)	1674 (0.3)	0.60

**Birth weight categorised using WHO definitions (34)*
Number in brackets indicates the percentage of all live births given PN in each category (Denominator data from ONS Birth characteristics in England and Wales)
Trend analysis for proportion of neonates admitted to a neonatal unit given PN in each category: p value was significant after Bonferroni-Holm correction for multiple comparisons for <1kg and <1.5kg categories
Neonates with missing data for birth weight = 4
†The ONS changed the way they clean birthweight data in 2017 and as a result the number of neonates born <1kg dropped from 3117 births in 2016 to 2538 in 2017 This change in the denominator inflated the proportion of <1kg births given PN in 2017.

Confidential: For Review Only
